# Supplementary material for: Suppression of angiopoietin-like 4 reprograms endothelial cell metabolism and inhibits angiogenesis
Source: Nat Commun. 2023 Dec 12;14:8251. doi: 10.1038/s41467-023-43900-0 (PMC10716292; doi:10.1038/s41467-023-43900-0)
Supplement: Supplementary file 1 — Supplementary Information [file 41467_2023_43900_MOESM1_ESM.pdf]

SUPPLEMENTARY INFORMATION

Suppression of Angiopoietin-like 4 Reprograms Endothelial Cell Metabolism and Inhibits Angiogenesis

Balkrishna Chaube[1,2,3], Kathryn M Citrin[1,2,3,4], Mahnaz Sahraei[1], Abhishek K. Singh[1,2], Diego Saenz de Urturi[1,2,3], Wen Ding[1], Richard W Pierce[2,5], Raaisa Raaisa[6], Rebecca Cardone[6], Richard Kibbey[3,4,6], Carlos Fernández-Hernando[1,2,3,7] and Yajaira Suárez[1,2,3,7*].

[1]Department of Comparative Medicine, Yale University School of Medicine, New Haven, CT, USA.

[2]Vascular Biology and Therapeutics Program, Yale University School of Medicine, New Haven, Connecticut, USA.

[3]Yale Center for Molecular and System Metabolism, Yale University School of Medicine, New Haven, Connecticut, USA.

[4]Department of Cellular & Molecular Physiology, Yale University, New Haven, CT, USA

[5]Department of Pediatrics, Yale University School of Medicine, New Haven, CT, USA.

[6]Department of Internal Medicine, Yale University, New Haven, CT, USA

[7]Department of Pathology, Yale University School of Medicine, New Haven, CT, USA.

Supplementary Figures 1-12 with legends

Supplementary Table 1-3

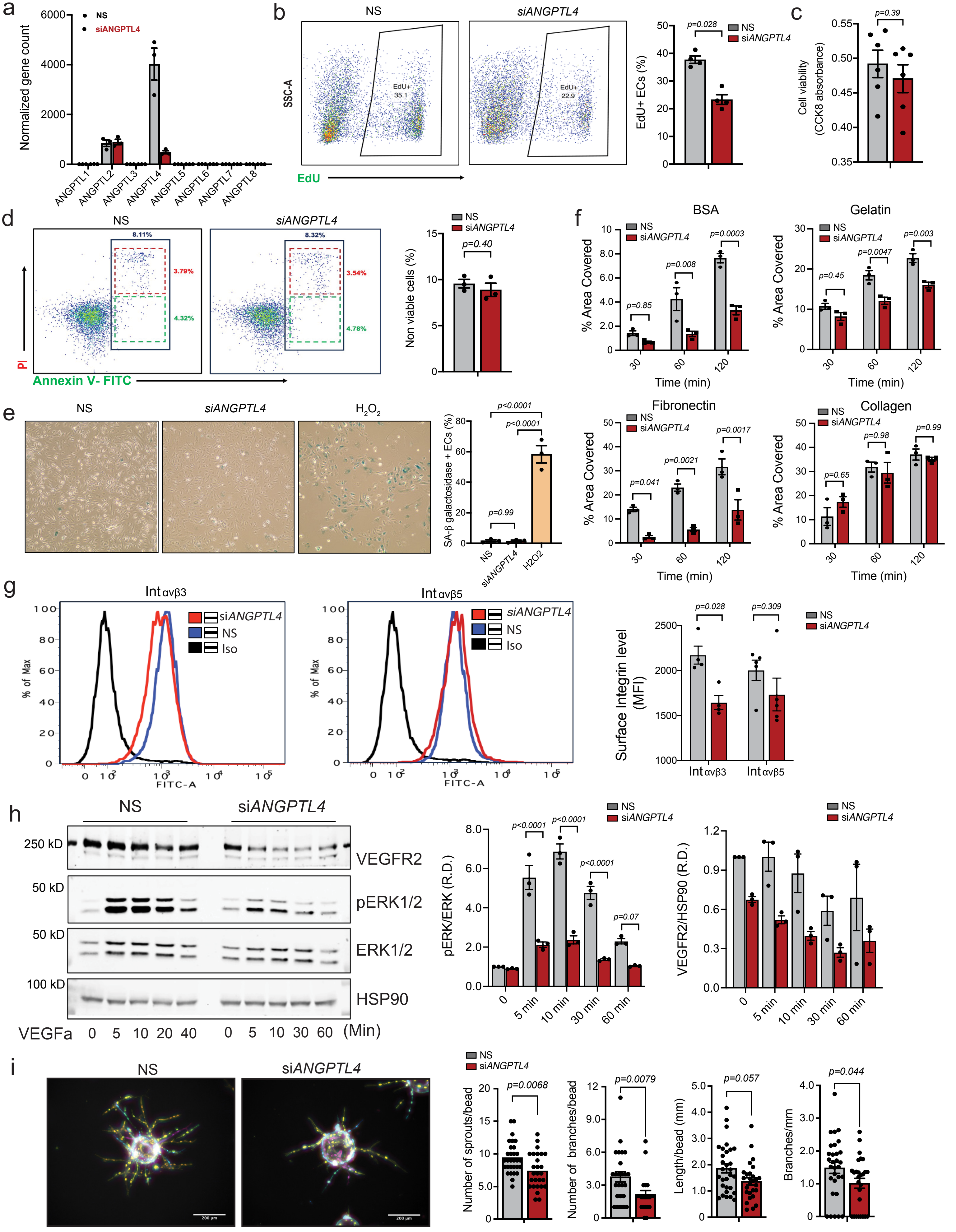

a
NS
siANGPTL4
Normalized gene count
6000
4000
2000
0
ANGPTL1
ANGPTL2
ANGPTL3
ANGPTL4
ANGPTL5
ANGPTL6
ANGPTL7
ANGPTL8
b
NS
siANGPTL4
SSC-A
EdU+
35.1
EdU+
22.9
EdU
p=0.028
NS
siANGPTL4
EdU+ ECs (%)
50
40
30
20
10
0
c
p=0.39
Cell viability
(CCK8 absorbance)
0.55
0.50
0.45
0.40
0.35
d
NS
siANGPTL4
8.11%
8.32%
3.79%
3.54%
4.32%
4.78%
PI
Annexin V- FITC
NS
siANGPTL4
p=0.40
Non viable cells (%)
15
10
5
0
f
BSA
Gelatin
NS
siANGPTL4
% Area Covered
p=0.85
p=0.008
p=0.0003
p=0.45
p=0.0047
p=0.003
Time (min)
30
60
120
Fibronectin
Collagen
p=0.041
p=0.0021
p=0.0017
p=0.65
p=0.98
p=0.99
e
NS
siANGPTL4
H2O2
p<0.0001
p<0.0001
p=0.99
SA-β galactosidase + ECs (%)
80
60
40
20
0
NS
siANGPTL4
H2O2
g
Int αvβ3
Int αvβ5
siANGPTL4
NS
Iso
% of Max
FITC-A
p=0.028
p=0.309
NS
siANGPTL4
Surface Integrin level
(MFI)
2500
2000
1500
1000
Int αvβ3
Int αvβ5
h
NS
siANGPTL4
250 kD
VEGFR2
50 kD
pERK1/2
50 kD
ERK1/2
100 kD
HSP90
VEGFa
0
5
10
20
40
0
5
10
30
60
(Min)
NS
siANGPTL4
p<0.0001
p<0.0001
p<0.0001
p=0.07
pERK/ERK (R.D.)
8.0
6.0
4.0
2.0
0.0
0
5 min
10 min
30 min
60 min
NS
siANGPTL4
VEGFR2/HSP90 (R.D.)
1.2
0.9
0.6
0.3
0.0
i
NS
siANGPTL4
NS
siANGPTL4
p=0.0068
Number of sprouts/bead
p=0.0079
Number of branches/bead
p=0.057
Length/bead (mm)
p=0.044
Branches/mm

Supplementary Fig. 1. ANGPTL4 in Required for EC Angiogenic Function *in vitro*. a, a-h, HUVECs were transfected for 6h with siRNA against *ANGPTL4* or NS control. Cells were harvested 60h post-transfection unless otherwise indicated. **a**, RNAseq analysis as shown in Fig. 1c (n=3 biological replicates). Normalized log2 expression of ANGPTL family genes in HUVECs. **b,** EdU incorporation in HUVECs assessed by flow cytometry at 48h post-transfection. Representative dot plot (left) and the bar graph showing the % EdU positive cells (right). **c,** At 60h post-transfection, cell viability was assessed by measuring the absorbance of CCK8 at 450nm. **d**, Apoptosis in HUVECs was assessed by staining with Annexin V and propidium iodide (PI), by flow cytometry. Right panel displays the percentage of total non-viable cells, which is the sum of both Annexin V+ and PI-Annexin V double-positive cells, for each group. **e**, Senescence-associated β-galactosidase staining was conducted. HUVECs treated with 100 μM H_2O_2 used as a positive control. Representative images show the β-galactosidase staining (left). Rght panel displays the quantification of β-galactosidase positive cells. **f**, Cell adhesion was evaluated on glass coverslip coated with indicated matrix protein at the indicated time points. Bar graphs represents %area covered on glass coverslips. **g,** Representative histograms for integrins $\alpha v\beta 3$ and $\alpha v\beta 5$ surface protein levels as accessed by flow cytometry (Left), M.F.I was used for quantification on the right. (**b-g**) n=3-6 independent experiments as shown, performed in duplicate. **h**, Representative immunoblot images showing the level of VEGFR2, pERK1/2 and ERK1/2. HSP90 was used as a loading control. Right panel shows the densitometric quantitation of indicated immunoblots (Representative blots of 1 experiment out of 3 with similar results). **i,** Representative micrograph showing the 3-D HUVECs sprouting in fibrin gels. Right panels showing the quantitation of different parameters as indicated (Data representative of a single experiment. The dots represent the mean quantification derived from four images per technical replicate). Data are represented as means ± SEM. Mann–Whitney U test in (**b**-**d**) (**e**) (**g**) and (**i**). One-way and two way ANOVA with Tukey's multiple comparisons test in (**e**) and (**f**) respectively. Scale bar, 200 μm (**i**). Exact *p* values were shown for each comparison. Source data are provided as a Source data file.

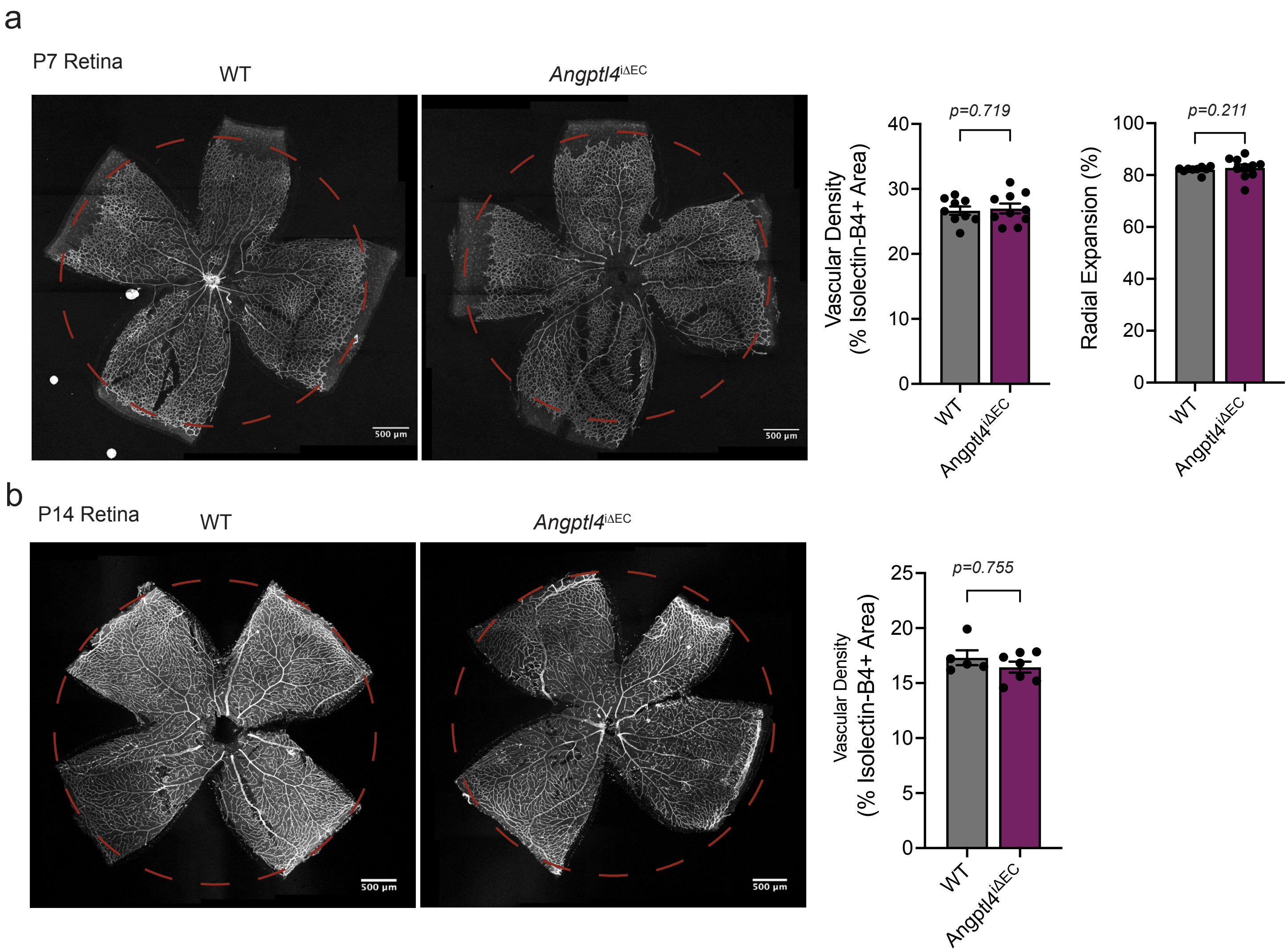
a
P7 Retina
WT
Angptl4iΔEC
500 µm
500 µm
p=0.719
Vascular Density
(% Isolectin-B4+ Area)
40
30
20
10
0
WT
Angptl4iΔEC
p=0.211
Radial Expansion (%)
100
80
60
40
20
0
WT
Angptl4iΔEC
b
P14 Retina
WT
Angptl4iΔEC
500 µm
500 µm
p=0.755
Vascular Density
(% Isolectin-B4+ Area)
25
20
15
10
5
0
WT
Angptl4iΔEC

Supplementary Fig. 2. EC specific deletion of *Angptl4* does not affect physiological retinal angiogenesis. *Angptl4* deletion on retinal vasculature (mice injected with tamoxifen P1–P3, assessed at P7 and P14). **a**, Retinal whole mounts. Left**,** representative immunofluorescence of IB4-AF647 staining (grey) in P7 mouse retinas showing no apparent change in vascular density or radial expansion in *Angptl4*$^{i\Delta EC}$ mice (n= 10) as compared to WT mice (n= 9). Right, quantification of vascular density and radial expansion (n= 10 or 9 per genotype, atleast 4 images per mouse was quantified). **b**, Retinal whole mounts. Left, representative immunofluorescence staining of IB4-AF647 (grey) in P14 mouse retinas showing no apparent change in vascular density in *Angptl4*$^{i\Delta EC}$ mice (n= 7) as compared to WT mice (n= 5). Right, quantification of vascular density (n= 7or 5 per genotype, atleast 4 images per mouse was quantified). All data are represented as means ± SEM. Student's t test (two-tailed, unpaired, nonparametric) in (**a** and **b**). Scale bars, 500 μm. Exact *p* values were shown for each comparison. Source data are provided as a Source data file.

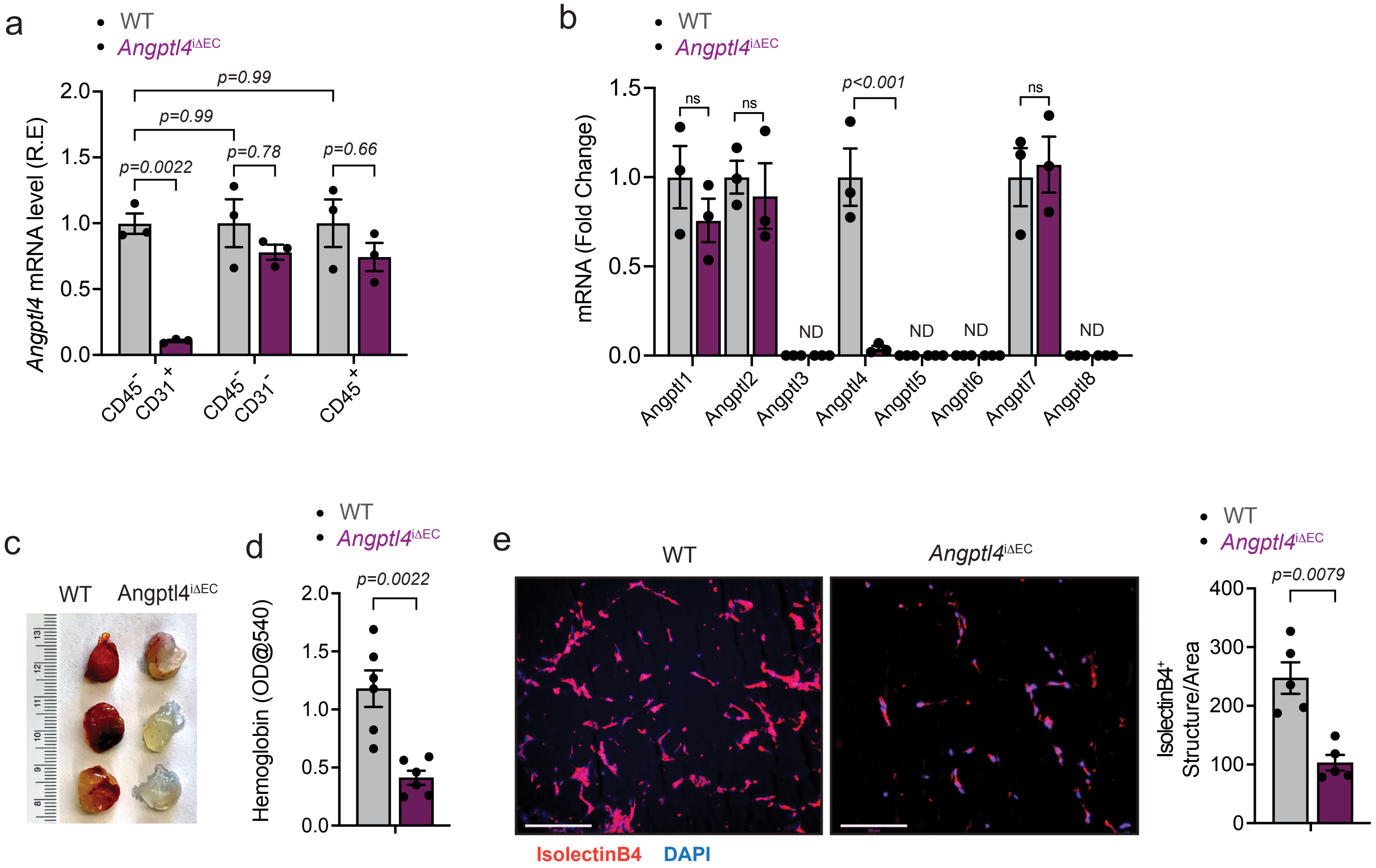
a
WT
Angptl4iΔEC
Angptl4 mRNA level (R.E)
p=0.99
p=0.99
p=0.0022
p=0.78
p=0.66
CD45- CD31+
CD45- CD31-
CD45+
b
WT
Angptl4iΔEC
mRNA (Fold Change)
ns
ns
p<0.001
ns
ND
Angptl1
Angptl2
Angptl3
Angptl4
Angptl5
Angptl6
Angptl7
Angptl8
c
WT
Angptl4iΔEC
d
Hemoglobin (OD@540)
p=0.0022
e
WT
Angptl4iΔEC
IsolectinB4
DAPI
IsolectinB4+ Structure/Area
p=0.0079

Supplementary Figure 3: **EC specific deletion of *Angptl4* Impairs angiogenesis. a,** Bar graph showing the mRNA levels of *Angptl4* in different cells isolated from lungs of WT and *Angptl4*$^{i\Delta EC}$ mice (n= 3 for each genotype) via FACS sorting. **b**, qRT-PCR analysis of *Angptl* family genes in MLECs isolated from WT and *Angptl4*$^{i\Delta EC}$ mice (n= 3 for each genotype). **c,** Representative images showing 3 Matrigel plugs isolated from WT and *Angptl4*$^{i\Delta EC}$ mice. **d**, Quantification of hemoglobin content in the Matrigel plugs. (n=6 per genotype). **e,** Left, representative images of Matrigel plugs sections stained with isolectin B4. Right, quantitation of EC positive area/vessel area in the plugs (n=5 per genotype). Scale bars, 125μm (**e**) Data are represented as means ± SEM. One-way ANOVA with Tukey's multiple comparisons test in (**a**) Student's t test (two-tailed, unpaired, nonparametric) in **(b**) and Mann–Whitney U test **(d** and **e**). Exact *p* values were shown for each comparison. Source data are provided as a Source data file.

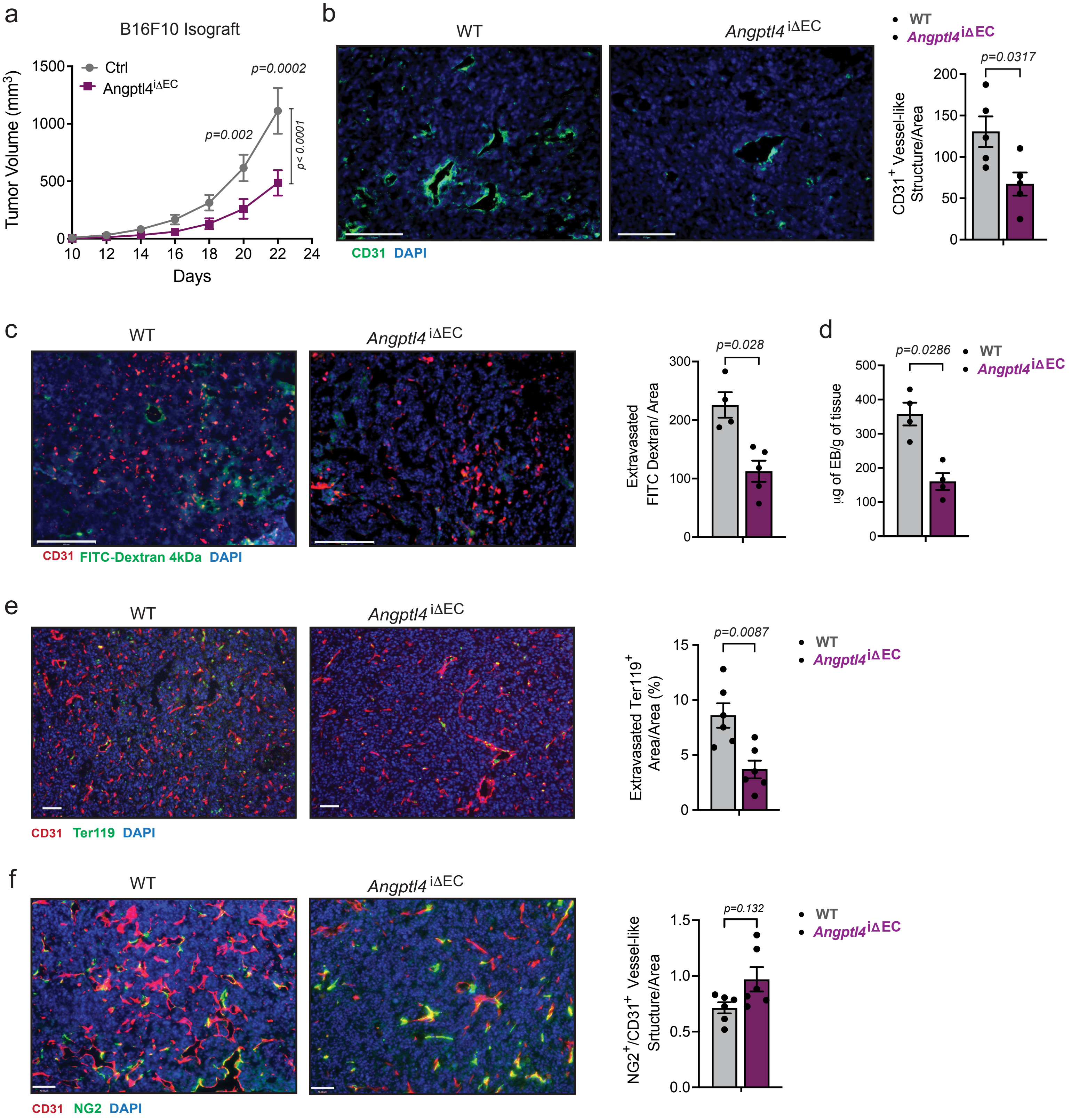

a
B16F10 Isograft
Ctrl
Angptl4$^{i\Delta EC}$
Tumor Volume (mm^3)
Days
p=0.002
p=0.0002
p< 0.0001
b
WT
Angptl4 iΔEC
CD31 DAPI
WT
Angptl4$^{i\Delta EC}$
p=0.0317
CD31$^+$ Vessel-like Structure/Area
c
WT
Angptl4 iΔEC
CD31 FITC-Dextran 4kDa DAPI
p=0.028
Extravasated FITC Dextran/ Area
d
p=0.0286
WT
Angptl4 iΔEC
μg of EB/g of tissue
e
WT
Angptl4 iΔEC
CD31 Ter119 DAPI
p=0.0087
Extravasated Ter119$^+$ Area/Area (%)
WT
Angptl4 iΔEC
f
WT
Angptl4 iΔEC
CD31 NG2 DAPI
p=0.132
NG2$^+$/CD31$^+$ Vessel-like Srtucture/Area
WT
Angptl4 iΔEC

Supplementary Fig. 4. EC specific deletion of *Angptl4* Impairs tumour angiogenesis
a and **b,** Tumor growth analysis of WT and *Angptl4*$^{i\Delta EC}$ mice with s.c. injection of B16F10 cells in the dorsal flank (n=9 for each genotype). **a,** Tumor volume. **b,** Left, representative micrographs of CD31 (green) and DAPI immunostaining. Right, Quantification of CD31^{+} vessel-like structures. **c,** Left, representative images of cross-sections of LLC tumours from WT and *Angptl4*$^{i\Delta EC}$ mice stained for FITC-Dextran (4kDa) (green) and EC marker CD31 (red). Right, quantification of extravasated FITC-Dextran. **d,** LLC tumours bearing WT and *Angptl4*$^{i\Delta EC}$ mice were intravenously injected with EB. Bar graph represents the quantitation of extravasated EB extracted from the overnight incubation of tumours in formamide solution and absorbance was recorded at 580 nm (n=4 per genotype). **e,** Left, representative images of cross-sections of LLC tumours from WT and *Angptl4*$^{i\Delta EC}$ mice, stained for Ter119 (green) and EC marker CD31 (red). Right, quantification of extravasated Ter119. **f,** Left, representative micrographs of LLC tumours from WT and *Angptl4*$^{i\Delta EC}$ mice, stained for NG2 (green) and EC marker CD31 (red). Right, quantification of NG2 covered CD31^{+} vessel-like structures. Right panels (**b, c, e** and **f**) represent quantification of at least 4 different images from each mouse (**b**, n= 5 quantified per genotype out of 9 randomly selected, **c**, n=4 or 5 quantified per genotype. **e** and **f**, n=6 quantified per genotype out of 6 or 9 randomly selected). Scale bars, 125 μm (**b**), 200 μm (**c**), 70 μm (**e** and **f**). All data are represented as means ± SEM. Two-way ANOVA with Tukey's multiple comparisons test in (**a**) and Mann–Whitney U test in (**b-f**). Exact *p* values were shown for each comparison. Source data are provided as a Source data file.

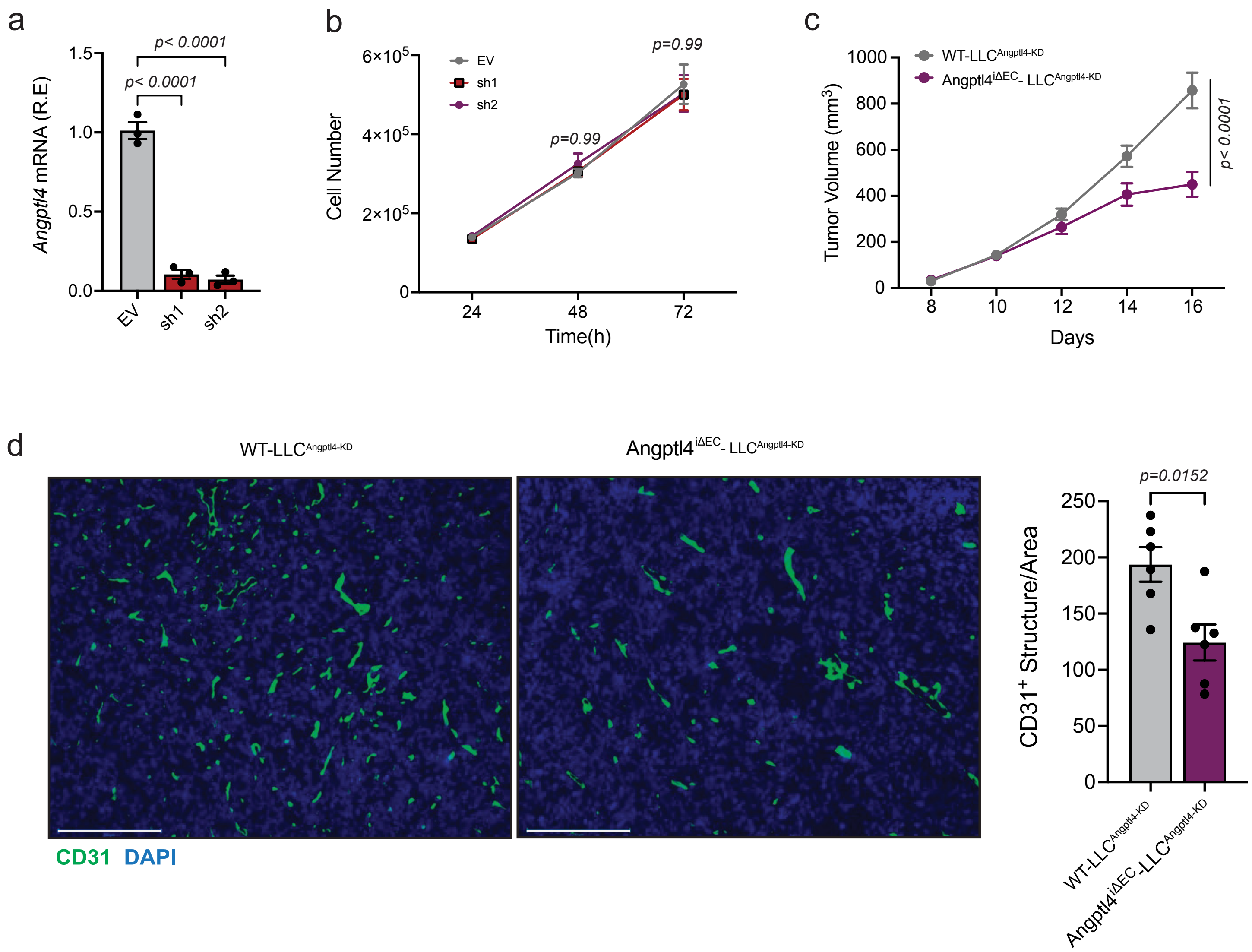
a
p< 0.0001
p< 0.0001
Angptl4 mRNA (R.E)
1.5
1.0
0.5
0.0
EV
sh1
sh2
b
6×10^5
4×10^5
2×10^5
0
EV
sh1
sh2
p=0.99
p=0.99
Cell Number
24
48
72
Time(h)
c
1000
800
600
400
200
0
WT-LLC Angptl4-KD
Angptl4 iΔEC - LLC Angptl4-KD
p< 0.0001
Tumor Volume (mm3)
8
10
12
14
16
Days
d
WT-LLC Angptl4-KD
Angptl4 iΔEC - LLC Angptl4-KD
CD31 DAPI
p=0.0152
250
200
150
100
50
0
CD31+ Structure/Area
WT-LLC Angptl4-KD
Angptl4 iΔEC -LLC Angptl4-KD

Supplementary Fig. 5. EC specific ANGPTL4 is required for endothelial tumour. a, LLCs were transduced with lentivirus carrying shRNAs against *Angptl4*, and stable clones were selected using puromycin. qRT-PCR data show reduced expression levels of *Angptl4* in LLC cells after achieving stable knockdown through lentiviral-mediated shRNA transduction (n= 3 independent experiments). **b,** Cell count analyses for LLC stable clones (EV vs. sh*Angptl4* referred to as LLC-$^{\text{Angptl4-KD}}$) reveal no effects on cell proliferation (n= 3 independent experiments). LLC clones with the highest knockdown efficiency (sh2) were selected for the tumor isograft analysis in WT and *Angptl4*$^{\text{i}\Delta\text{EC}}$ mice. **c,** Growth curve of LLC lacking ANGPTL4 (LLC-$^{\text{Angptl4-KD}}$) isograft in WT and *Angptl4*$^{\text{i}\Delta\text{EC}}$ mice (n= 6 for each genotype). **d**, Bar graph shows the end-stage tumour weight of LLC-$^{\text{Angptl4-KD}}$ derived tumours in WT and *Angptl4*$^{\text{i}\Delta\text{EC}}$ mice (n= 6 for each genotype). **e,** Left, representative micrographs of LLC-$^{\text{Angptl4-KD}}$ derived tumours in WT and *Angptl4*$^{\text{i}\Delta\text{EC}}$ mice, stained for CD31 (green) and DAPI (n= 6 for each genotype). Right panels represent quantification of at least 4 different images from each mouse (n=6 per genotype). Scale bars, 200 μm (**e**). All data are represented as means ± SEM. Mann–Whitney U test in (**d** and **e**). One way or Two-way ANOVA with Tukey's multiple comparisons test in (**a**) and (**b**, **c**) respectivily. Exact *p* values were shown for each comparison. Source data are provided as a Source data file.

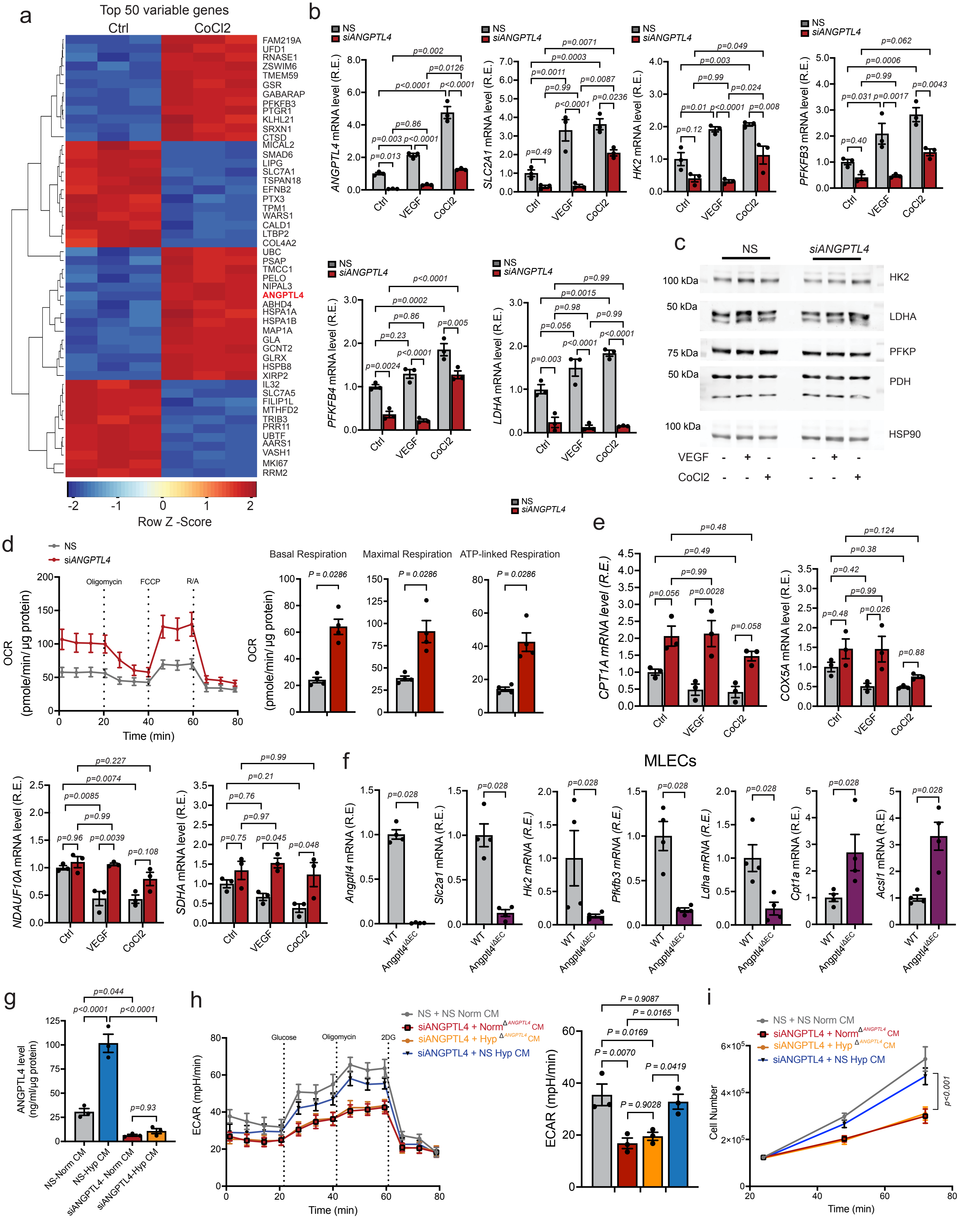

a
Top 50 variable genes
Ctrl
CoCl2
Row Z -Score
b
c
NS
siANGPTL4
HK2
LDHA
PFKP
PDH
HSP90
VEGF
CoCl2
d
Oligomycin
FCCP
R/A
Time (min)
Basal Respiration
Maximal Respiration
ATP-linked Respiration
e
f
MLECs
g
h
Glucose
2DG
NS + NS Norm CM
siANGPTL4 + NS Hyp CM
ECAR (mpH/min)
i
Cell Number

Supplementary Fig. 6. ANGPTL4 modulates VEGF or $CoCl_2$ induced metabolic response in HUVECs. HUVECs were transfected for 6h with siRNA against *ANGPTL4* or NS control and then treated with VEGF, $CoCl_2$ or vehicle control as indicated, cells were harvested 60h post-transfection. **a**, Heatmap representing the transcript levels of top 50 variable genes in HUVECs treated with 100 μM $CoCl_2$ from the RNAseq analysis, as shown in (Fig. 1c) (n=3 biological replicates). Color key: red, high correlation; blue, low correlation. **b,** HUVECs were transfected with siRNAs against NS or ANGPTL4 as described above. qRT-PCR analysis of indicated genes. Expression of these genes was normalized with 18S rRNA (n=3 independent experiments). **c,** Immunoblots showing the protein levels of glycolytic enzymes in HUVECs grown under similar conditions as used in (**b**). (Immunoblots represent a single experiment). **d,** Seahorse analysis showing Oxygen consumption rate (OCR). HUVECs were transfected as described above. 48h post-transfection, they were replated on Seahorse plates. Right panel represents the quantification of basal, maximal and ATP linked respiration (n=4 independent experiments). **e,** qRT-PCR analysis of indicated genes related to FAO in HUVECs grown under similar conditions as in (**b**) (n= 3 independent experiments perfomed in duplicates). **f,** qRT-PCR analysis of indicated genes related to glycolysis and FAO in MLECs isolated from WT and *Angptl4*$^{i\Delta EC}$ mice (n= 4 for each genotype). Expression of these genes was normalized with 18S rRNA. **g**, HUVECs were transfected with siRNA against *ANGPTL4* as described above and grown in either normoxia or hypoxia for 48h. Quantitative ELISA analysis of secreted levels of ANGPTL4 in the CM (n= 3 independent experiments perfomed in duplicates). **h**, (Left panel) Seahorse analysis in *ANGPTL4* KD HUVECs grown in the presence of CM enriched (NS-Hyp CM) or depleted (*siANGPTL4*-Hyp CM refers to as Hyp$^{\Delta ANGPTL4}$ CM) for ANGPTL4. Right panel shows the quantification of ECAR (glycolysis) (n=3 independent experiments). **i**, Proliferation of *ANGPTL4* KD HUVECs grown in the presence of CM enriched or depleted for ANGPTL4, expressed as relative cell number to NS at indicated time points (n=3 independent experiments). Unless indicated above, all data are represented as means ± SEM. Mann–Whitney U test in (**d** and **f**). Two-way ANOVA with Tukey's multiple comparisons test in (**b**, **e**, and **g**-**i**). Hyp- Hypoxia, Norm-Normoxic condition, CM-Conditioned Medium. Exact *p* values were shown for each comparison. Source data are provided as a Source data file.

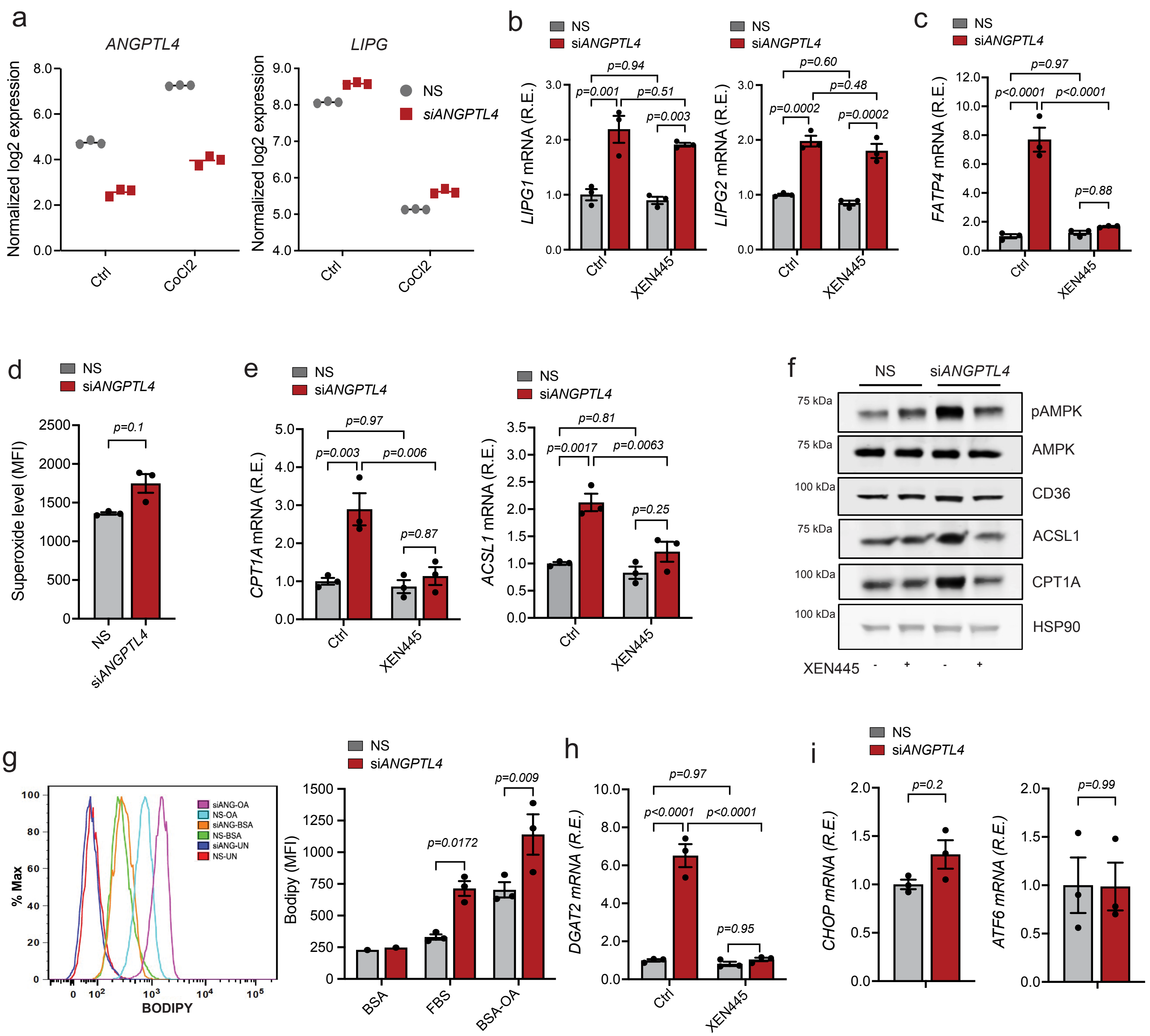

a
ANGPTL4
Normalized log2 expression
8.0
6.0
4.0
2.0
0.0
Ctrl
CoCl2
LIPG
Normalized log2 expression
9.0
8.0
7.0
6.0
5.0
4.0
NS
siANGPTL4
b
NS
siANGPTL4
LIPG1 mRNA (R.E.)
p=0.94
p=0.001
p=0.51
p=0.003
XEN445
LIPG2 mRNA (R.E.)
p=0.60
p=0.48
p=0.0002
p=0.0002
c
FATP4 mRNA (R.E.)
p=0.97
p<0.0001
p<0.0001
p=0.88
d
Superoxide level (MFI)
p=0.1
e
CPT1A mRNA (R.E.)
p=0.97
p=0.003
p=0.006
p=0.87
ACSL1 mRNA (R.E.)
p=0.81
p=0.0017
p=0.0063
p=0.25
f
NS
siANGPTL4
75 kDa
pAMPK
75 kDa
AMPK
100 kDa
CD36
75 kDa
ACSL1
100 kDa
CPT1A
100 kDa
HSP90
XEN445 - + - +
g
siANG-OA
NS-OA
siANG-BSA
NS-BSA
siANG-UN
NS-UN
% Max
BODIPY
Bodipy (MFI)
p=0.0172
p=0.009
BSA
FBS
BSA-OA
h
DGAT2 mRNA (R.E.)
p=0.97
p<0.0001
p<0.0001
p=0.95
i
CHOP mRNA (R.E.)
p=0.2
ATF6 mRNA (R.E.)
p=0.99

Supplementary Fig. 7. ANGPTL4 regulates EC lipase activity and FA metabolism. **a,** **a**, RNAseq analysis showing the normalized log2 expression of *ANGPTL4* and *LIPG* in HUVECs as in (Fig 3a) (n=3 biological replicates). **b** and **c,** HUVECs were transfected for 6h with siRNA against *ANGPTL4* or NS control and then treated with XEN445 or vehicle control, cells were harvested 60h post-transfection. qRT-PCR analysis of showing expression of *LIPG1, LIPG2* genes (**b**) and *FATP4* (**c**). Expression of these genes was normalized with 18S rRNA (**b** and **c,** n= 3 independent experiments as shown, performed in duplicate). **d,** Superoxide level in HUVECs post 60h transfection with *ANGPTL4* siRNA as determined by mean fluorescence intensity (MFI) of DHE (dihydroethidium) via Flow cytometry (n=3 independent experiments). **e,** qRT-PCR analysis of *CPT1A and ACSL1* genes in HUVECs grown under the conditions described above. Expression of these genes was normalized with 18S rRNA (n=3 independent experiments). **f,** Immunoblots of indicated molecules in whole cell lysate of HUVECs grown under the conditions described above. HSP90 was used as loading control (Immunoblots represent a single experiment). **g,** HUCECs were transfected as described above. 60h post-transfection, HUVECs were FBS-starved for 12h followed by loading with either BSA, 20% FBS or BSA-Oleic acid for 6h. Left, representative histogram showing the neutral lipid storage in HUVECs as determined by staining with BODIPY via Flow cytometry. Right, M.F.I was used for quantification on the right. (n=3 independent experiments). **h,** mRNA level of *DGAT2* gene in HUVECs grown under the similar conditions used in (**b**). Expression of *DGAT2* gene was normalized with 18S. **i,** mRNA levels of ER stress marker genes in HUVECs grown under the similar conditions used in (**d**) (n=3 independent experiments performed in triplicate). All data are represented as means ± SEM. Two-way ANOVA with Tukey's multiple comparisons test in (**b, c, e**, **g** and **h**). Mann–Whitney U test in (**d** and **i**). Exact *p* values were shown for each comparison. Source data are provided as a Source data file.

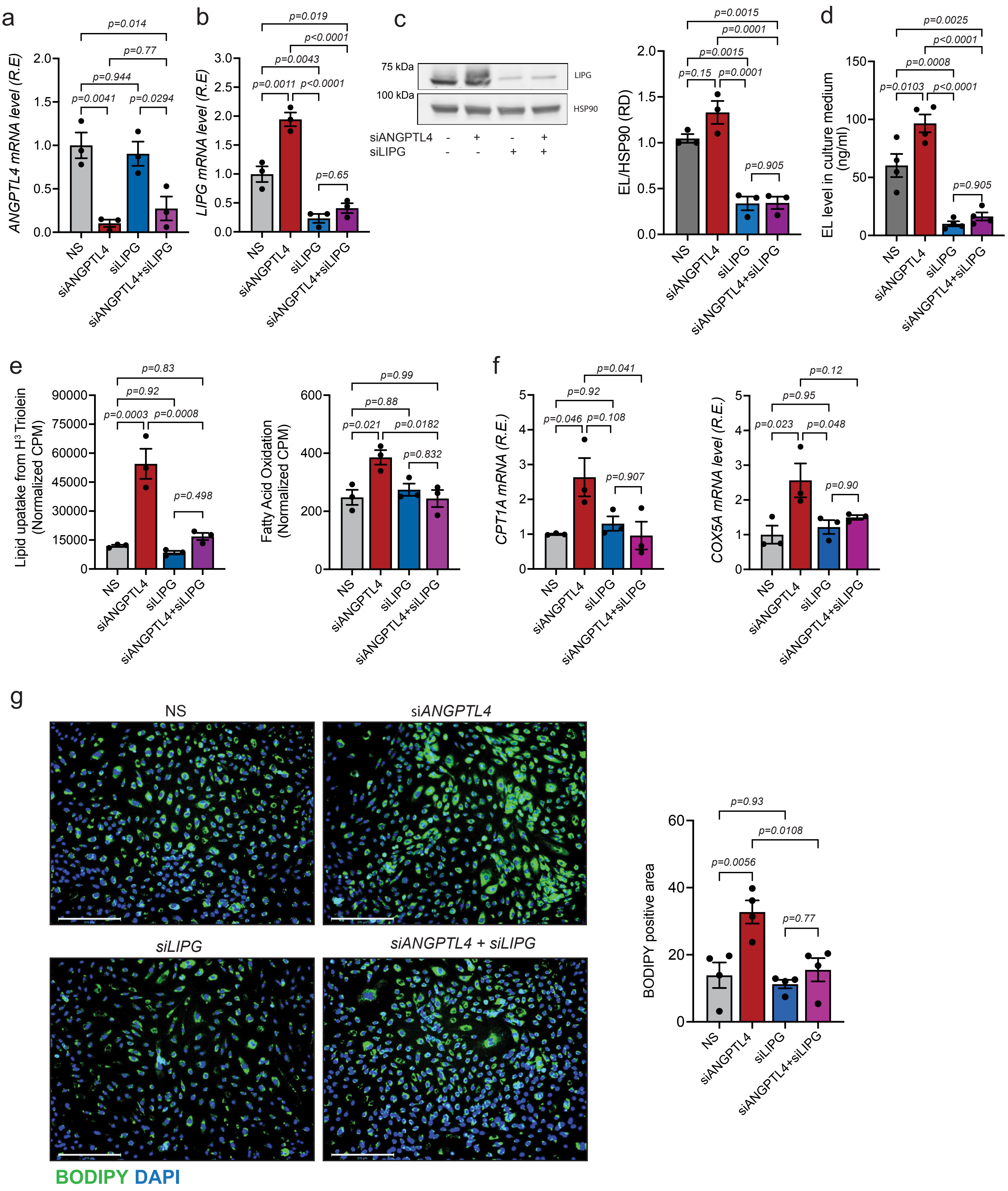

a
ANGPTL4 mRNA level (R.E)
p=0.014
p=0.77
p=0.944
p=0.0041
p=0.0294
NS
siANGPTL4
siLIPG
siANGPTL4+siLIPG
b
LIPG mRNA level (R.E)
p=0.019
p<0.0001
p=0.0043
p=0.0011
p<0.0001
p=0.65
c
75 kDa
100 kDa
LIPG
HSP90
siANGPTL4
siLIPG
EL/HSP90 (RD)
p=0.0015
p=0.0001
p=0.0015
p=0.15
p=0.0001
p=0.905
d
EL level in culture medium (ng/ml)
p=0.0025
p<0.0001
p=0.0008
p=0.0103
p<0.0001
p=0.905
e
Lipid upatake from H³ Triolein (Normalized CPM)
p=0.83
p=0.92
p=0.0003
p=0.0008
p=0.498
Fatty Acid Oxidation (Normalized CPM)
p=0.99
p=0.88
p=0.021
p=0.0182
p=0.832
f
CPT1A mRNA (R.E.)
p=0.041
p=0.92
p=0.046
p=0.108
p=0.907
COX5A mRNA level (R.E.)
p=0.12
p=0.95
p=0.023
p=0.048
p=0.90
g
NS
siANGPTL4
siLIPG
siANGPTL4 + siLIPG
BODIPY DAPI
BODIPY positive area
p=0.93
p=0.0108
p=0.0056
p=0.77

Supplementary Fig. 8. ECs lacking *ANGPTL4* exhibit elevated FA uptake and storage in lipase dependent manner. a-e, HUVECs were co-transfected with siRNAs against ANGPTL4 and/or LIPG, or with a NS control, for 6h. Cells were then harvested 60h post-transfection. **a** and **b**, mRNA levels of *ANGPTL4* and *LIPG* showing the knockdown efficiency of these genes (n= 3 independent experiment). **c,** Representative immunoblots of EL in whole cell lysate of HUVECs grown under the conditions described above (Representative blots of 1 experiment out of 3 with similar results). HSP90 was used as loading control. Right panel shows the densitometric quantitation of immunoblots (n=3 independent experiments). **d**, Bargraph shows the secreted level of EL in the heparinized culture medium from the HUVECs grown in the similar conditions as used in (**a**), determined by quantitative ELISA (n= 4 independent experiment). **e**, Lipid uptake from [^{3}H]-triolein in HUVECs under the similar growth condition used in (**a**). Right panel shows FAO as determined by oxidation of C^{14}-Palmitate (n= 3 independent experiment). **f**, qRT-PCR analysis of *CPT1A and COX5A* genes in HUVECs grown under similar conditions used in (**a**) (n= 3 independent experiment). **g**, Representative micrograph showing the neutral lipid accumulation in HUVECs stained with BODIPY (Representative experiment out of 2 with similar results, dots are the mean of the quantification of 4 images per technical replicates). Scale bars, 275 μm (**g**). All data are represented as means ± SEM. Two-way ANOVA with Tukey's multiple comparisons test in (**a**-**g**). Exact *p* values were shown for each comparison. Source data are provided as a Source data file.

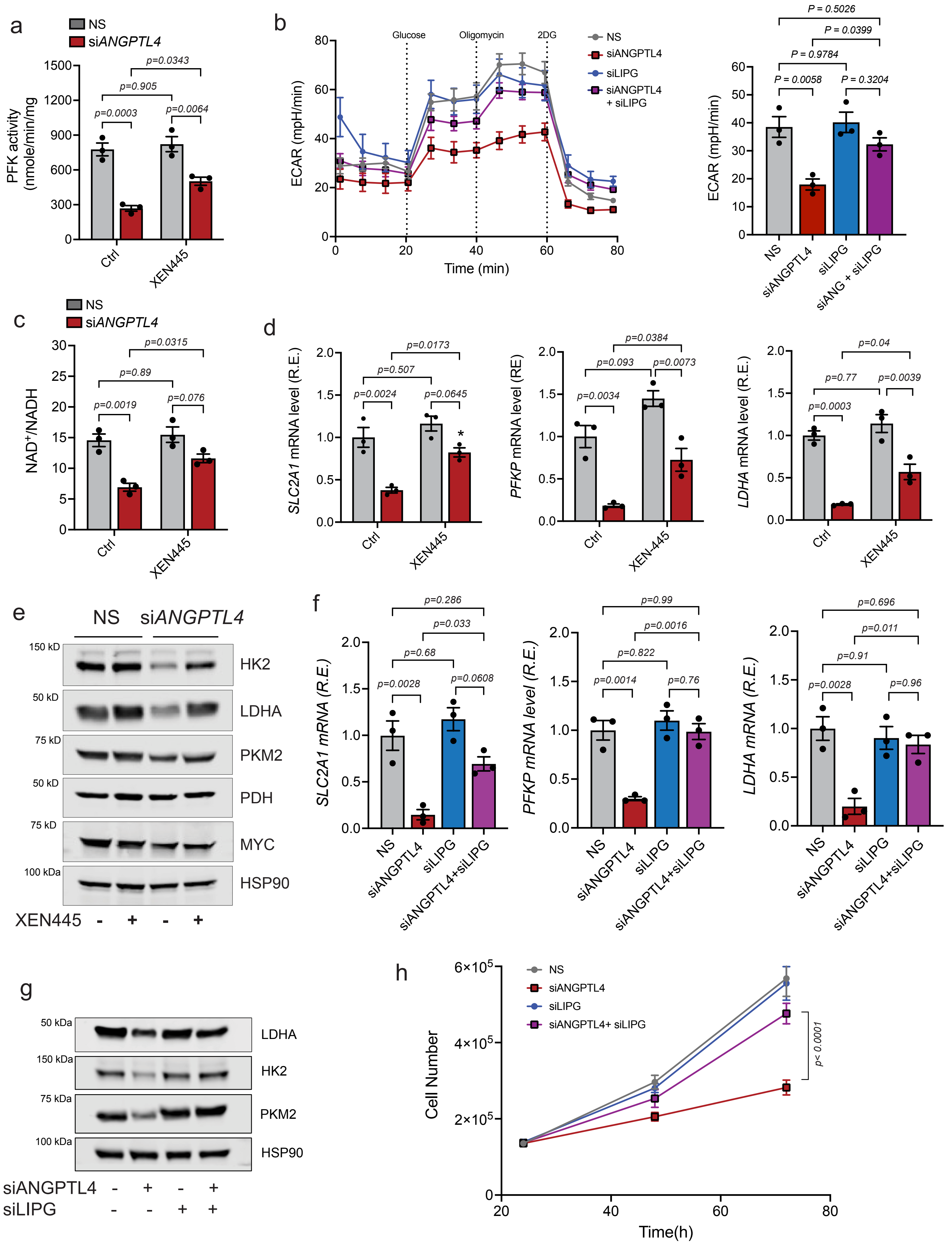

a
NS
siANGPTL4
PFK activity (nmole/min/mg
p=0.0343
p=0.905
p=0.0003
p=0.0064
Ctrl
XEN445
b
Glucose
Oligomycin
2DG
ECAR (mpH/min)
Time (min)
NS
siANGPTL4
siLIPG
siANGPTL4 + siLIPG
P = 0.5026
P = 0.0399
P = 0.9784
P = 0.0058
P = 0.3204
siANG + siLIPG
c
NAD+/NADH
p=0.0315
p=0.89
p=0.0019
p=0.076
d
SLC2A1 mRNA level (R.E.)
p=0.0173
p=0.507
p=0.0024
p=0.0645
PFKP mRNA level (RE)
p=0.0384
p=0.093
p=0.0073
p=0.0034
XEN-445
LDHA mRNA level (R.E.)
p=0.04
p=0.77
p=0.0039
p=0.0003
e
150 kD
50 kD
75 kD
100 kDa
HK2
LDHA
PKM2
PDH
MYC
HSP90
f
SLC2A1 mRNA (R.E.)
p=0.286
p=0.033
p=0.68
p=0.0028
p=0.0608
siANGPTL4+siLIPG
PFKP mRNA level (R.E.)
p=0.99
p=0.0016
p=0.822
p=0.0014
p=0.76
LDHA mRNA (R.E.)
p=0.696
p=0.011
p=0.91
p=0.0028
p=0.96
g
50 kDa
150 kDa
75 kDa
h
Cell Number
Time(h)
siANGPTL4+ siLIPG
p< 0.0001

Supplementary Fig. 9. *ANGPTL4* knockdown in HUVECs rewire glucose metabolism via activating LIPG. a, HUVECs were transfected for 6h with siRNA against *ANGPTL4* or NS control and then treated with XEN445 or vehicle control, cells were harvested 60h post-transfection. Enzyme activity of PFK (n= 3 independent experiment performed in duplicates. **b,** HUVECs were co-transfected with siRNAs against ANGPTL4 and/or LIPG, or with a NS control, for 6h. 48h post-transfection, they were replated on Seahorse plates. Seahorse analysis evaluates the extracellular acidification rate (glycolysis). Bar graph on the right is quantification of extracellular acidification rate (n=3 independent experiments). **c,** Determination of NAD^+/NADH in HUVECs upon silencing *ANGPTL4* as described in (**a**) (n= 3 independent experiment performed in triplicates). **d**, qRT-PCR analysis of glycolytic metabolism genes in HUVECs grown under similar conditions as in (**a**). Expression of these genes was normalized with 18S (n= 3 independent experiment performed in duplicates). **e,** Representative immunoblots showing the protein levels of glycolytic enzymes in HUVECs grown under similar conditions as in (**a**). (Representative blots of 1 experiment out of 3 with similar results). **f**, qRT-PCR analysis of glycolytic metabolism genes in HUVECs grown under similar conditions used in (**b**). Expression of these genes was normalized with 18S (n= 3 independent experiment performed in duplicates). **g,** Immunoblots showing the protein levels of glycolytic enzymes in HUVECs (Immunoblots represent a single experiment). **h,** Proliferation of HUVECs grown under similar conditions used in (**b**), expressed relative cell number to NS at indicated time points post-transfection (n= 3 independent experiment). All data are represented as means ± SEM. Two-way ANOVA with Tukey's multiple comparisons test in (**a**-**h**). Exact *p* values were shown for each comparison. Source data are provided as a Source data file.

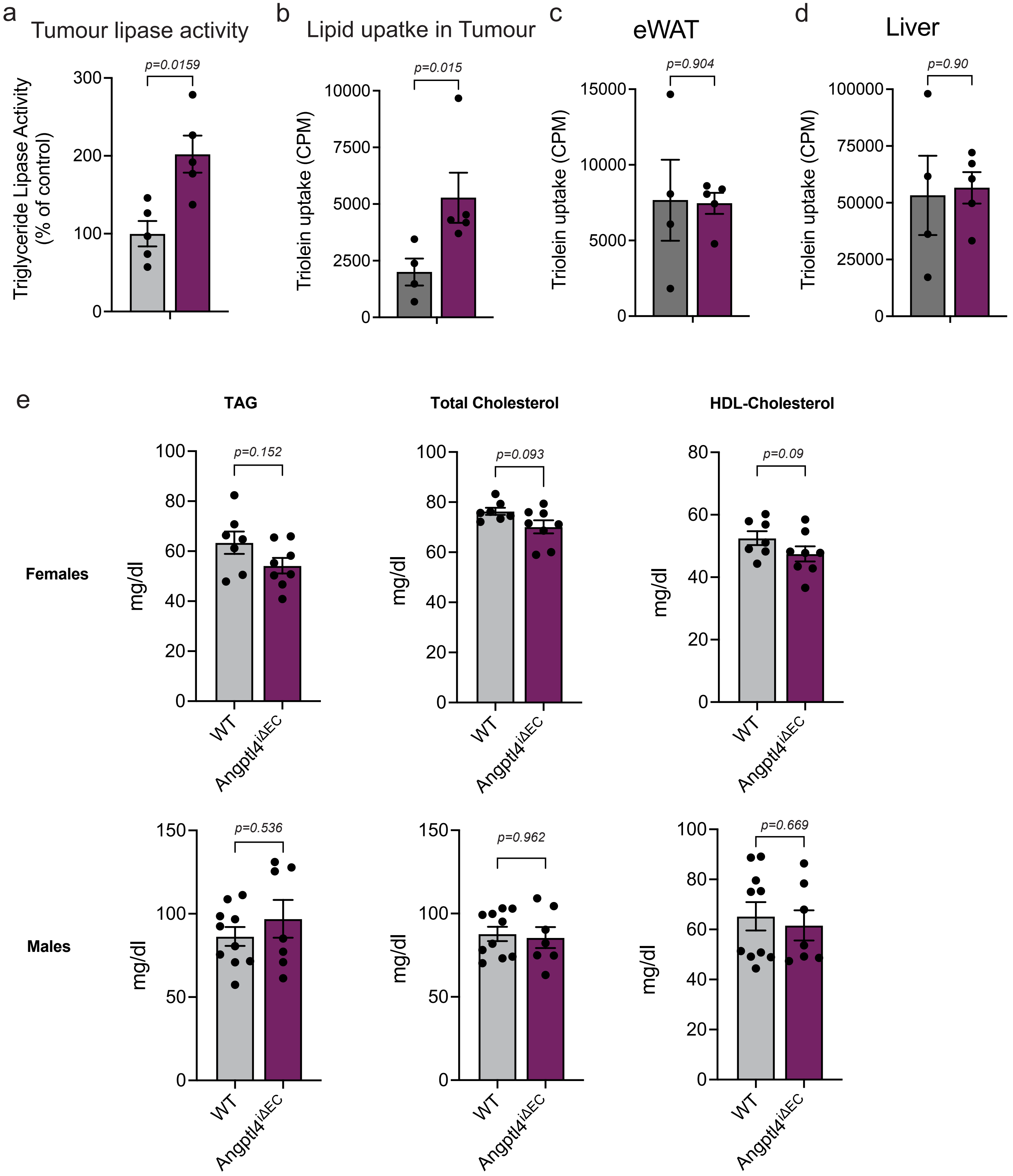

a
Tumour lipase activity
Triglyceride Lipase Activity (% of control)
p=0.0159
b
Lipid upatke in Tumour
Triolein uptake (CPM)
p=0.015
c
eWAT
Triolein uptake (CPM)
p=0.904
d
Liver
Triolein uptake (CPM)
p=0.90
e
TAG
Total Cholesterol
HDL-Cholesterol
Females
Males
mg/dl
p=0.152
p=0.093
p=0.09
p=0.536
p=0.962
p=0.669
WT
Angptl4iΔEC

Supplementary Fig. 10. EC specific deletion of *Angptl4* does not affect plamsa lipid levels *in vivo*. a, Triglyceride lipase activity in the tumour from WT and *Angptl4*$^{i\Delta EC}$ mice. **b-d,** Lipid uptake in the tumour (**b**), eWAT (**c**), and liver (**d**) from [^{3}H]-triolein in WT and *Angptl4*$^{i\Delta EC}$ mice (n= 4-5 mice per genotype). **e,** Levels of triglycerides (TAG), total cholesterol and HDL cholesterol in the plasma from females (upar panel) and males (lower panel) WT and *Angptl4*$^{i\Delta EC}$ mice (females n= 7-8 mice in each group; males n= 7-10 mice in each group). All data are represented as means ± SEM. Mann–Whitney U test in (**a**-**e**). Exact *p* values were shown for each comparison. Source data are provided as a Source data file.

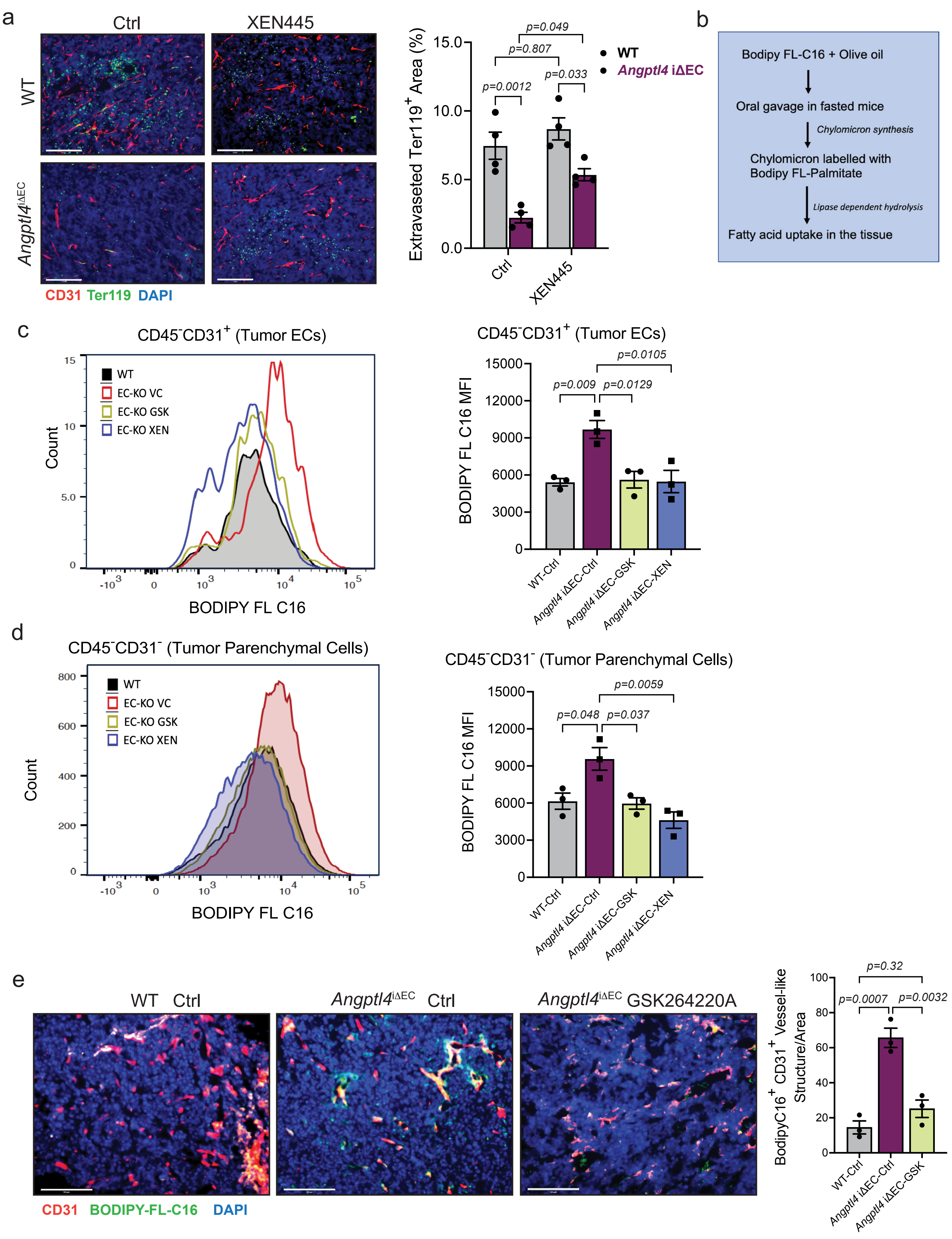
a
Ctrl
XEN445
WT
Angptl4iΔEC
CD31 Ter119 DAPI
Extravaseted Ter119+ Area (%)
p=0.049
p=0.807
p=0.0012
p=0.033
WT
Angptl4 iΔEC
Ctrl
XEN445
b
Bodipy FL-C16 + Olive oil
Oral gavage in fasted mice
Chylomicron synthesis
Chylomicron labelled with Bodipy FL-Palmitate
Lipase dependent hydrolysis
Fatty acid uptake in the tissue
c
CD45-CD31+ (Tumor ECs)
WT
EC-KO VC
EC-KO GSK
EC-KO XEN
Count
BODIPY FL C16
BODIPY FL C16 MFI
p=0.0105
p=0.009
p=0.0129
WT-Ctrl
Angptl4 iΔEC-Ctrl
Angptl4 iΔEC-GSK
Angptl4 iΔEC-XEN
d
CD45-CD31- (Tumor Parenchymal Cells)
p=0.0059
p=0.048
p=0.037
e
WT Ctrl
Angptl4iΔEC Ctrl
Angptl4iΔEC GSK264220A
CD31 BODIPY-FL-C16 DAPI
BodipyC16+ CD31+ Vessel-like Structure/Area
p=0.32
p=0.0007
p=0.0032
WT-Ctrl
Angptl4 iΔEC-Ctrl
Angptl4 iΔEC-GSK

Supplementary Fig. 11. EC specific ANGPTL4 regulates vascular lipolysis *in vivo* via inhibiting. Tumor analysis of WT Ctrl (n=4), WT XEN445 treated (n=4), *Angptl4*$^{i\Delta EC}$ Ctrl (n=4) mice and *Angptl4*$^{i\Delta EC}$ XEN445 treated (n=4) with s.c. injection of LLCs in the dorsal flank as described in (Fig. 2) **a,** Left, representative micrographs Ter119 (green) and CD31 (red) immunostaining. Right panel represent quantification of extravascular Ter119 in tumours.(n=4 quantified per genotype, at least 4 different images from each mouse were quantified). **b,** Scheme showing the experimental strategy. **c** and **d**, Flow cytometry analysis of BODIPY-FL-C16 uptake in tumour parenchymal ($CD45^-$ $CD31^-$ cells) (**c**), and tumour ECs ($CD45^-$ $CD31^+$) (**d**) from WT and *Angptl4*$^{i\Delta EC}$ treated with or without XEN445 or GSK264220A (n= 3 mice per group). Right panels, MFI of BODIPY-FL-C16 is quantified for the indicated groups. **e,** Left, representative micrographs of LLC tumours from WT and *Angptl4*$^{i\Delta EC}$ mice treated with GSK264220A, stained for EC marker CD31 (red) and fluorescent FA (BODIPY-FL-C16) (green). Right panel represents quantitation of CD31 and BODIPY-FL-C16 double positive vessel like structures in tumours. (n= 3 mice per group, at least 4 different images from each mouse were quantified). Scale bars, 125 μm (**a** and **e**). All data are represented as means ± SEM. Two-way ANOVA with Tukey's multiple comparisons test in (**a**)**.** One-way ANOVA with Tukey's multiple comparisons test in (**c**-**e**). Exact *p* values were shown for each comparison. Source data are provided as a Source data file.

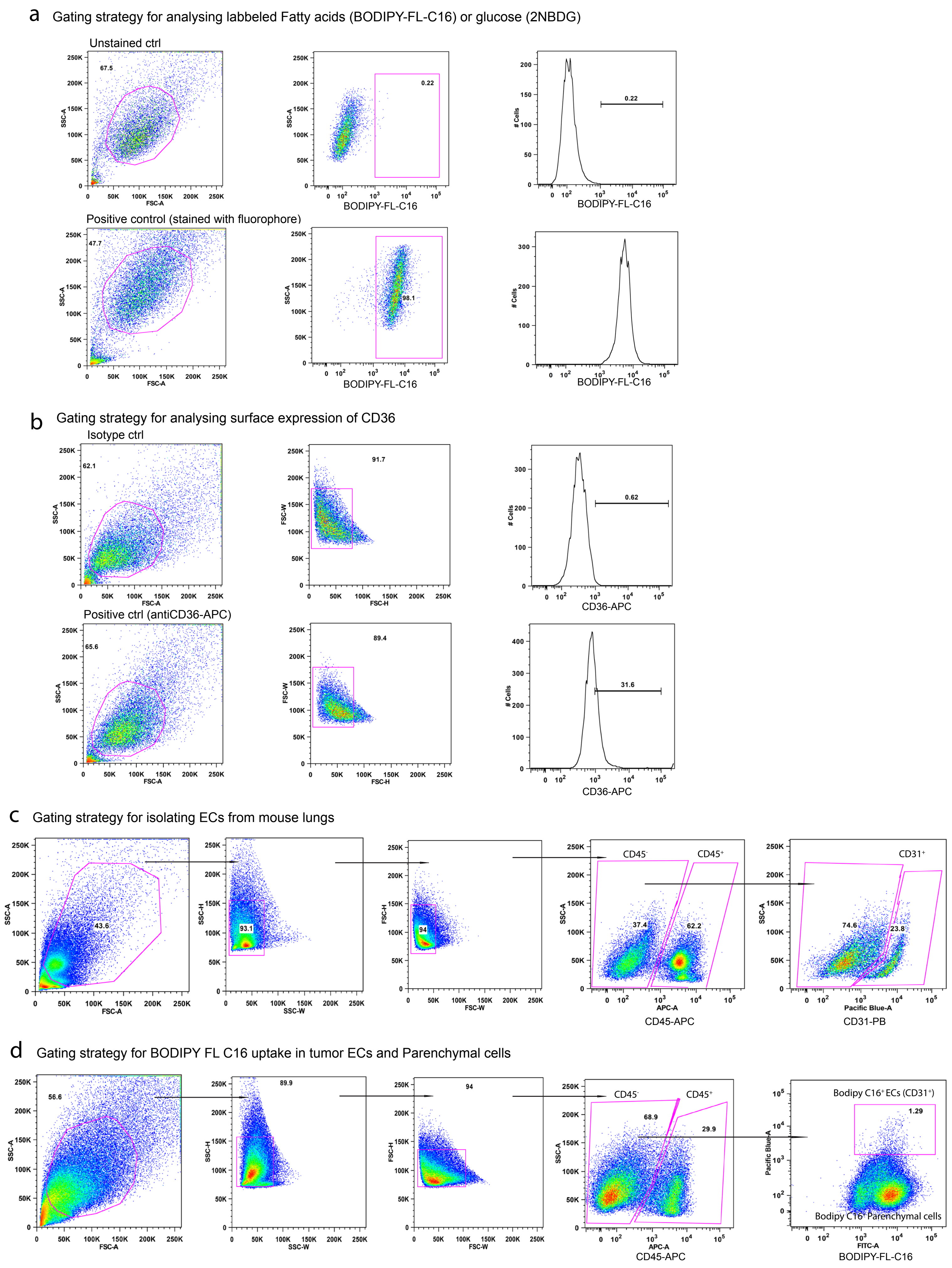
a Gating strategy for analysing labbeled Fatty acids (BODIPY-FL-C16) or glucose (2NBDG)
Unstained ctrl
67.5
0.22
0.22
SSC-A
FSC-A
BODIPY-FL-C16
Cells
Positive control (stained with fluorophore)
47.7
98.1
b Gating strategy for analysing surface expression of CD36
Isotype ctrl
62.1
91.7
0.62
FSC-W
FSC-H
CD36-APC
Positive ctrl (antiCD36-APC)
65.6
89.4
31.6
c Gating strategy for isolating ECs from mouse lungs
43.6
93.1
94
CD45-
CD45+
37.4
62.2
CD31+
74.6
23.8
SSC-H
SSC-W
APC-A
CD45-APC
Pacific Blue-A
CD31-PB
d Gating strategy for BODIPY FL C16 uptake in tumor ECs and Parenchymal cells
56.6
89.9
94
CD45-
CD45+
68.9
29.9
Bodipy C16+ ECs (CD31+)
1.29
Bodipy C16+ Parenchymal cells
FITC-A
BODIPY-FL-C16

Supplementary Fig. 12. a, Gating strategy for the FACS tests applied for the detection of fluorescently labbelled fatty acid (BODIPY-FL-C16) and glucose (2NBDG) in HUVECs as shown in Fig 4, Fig. 5, Fig. 6 and Supplementary Fig.7. **b**, Gating strategy for the FACS tests applied for the detection of surface expression of CD36 as shown in Fig. 5. **c**, Gating strategy for shorting ECs ($CD45^{-}CD31^{+}$) from the mouse lungs as shown in Supplementary Fig. 4. **d,** Gating strategy for the FACS tests applied for the detection of fluorescently labbelled fatty acid (BODIPY-FL-C16) in the tumor ECs and other parenchymal cells as shown in Supplementary Fig.11.

Supplementary Table 1: List of primers

Primers

Human qRT PCR Primers

**Gene name**	**Forward (5’-3’)**	**Reverse (5’-3’)**
**hANGPTL4**	CTCCACTTGGGACCAGGATCA	ATGGCTGCAGGTGCCAAAC
**hLIPG1**	CCACCCAATGGAAGGATTC	GCCATCACAGGAGACCAGTT
**hLIPG2**	GGCCTCTCAGTCTTGGTACAAC	CGGGGTTGAGACAGGTAGC
**hHK2**	ATCCCCCAGCACTGATGTT	TCACATCCTCAGTGGAGACCT
**hPFKFB3**	TCTCTCCTCCCACCTGGAC	GGTCACACTTCTGCCATCAA
**hPFKFB4**	CTTCAGGCCTGCTCTCACA	CTCGAGCACTTCGGTGTGT
**hLDHA**	TTTTCCCAGTGAGTCACATCC	TTGGAAGAATTATGCACAAGACA
**hSLC2A1**	CAGATGATGCGGGAGAAGA	ACAGCTGCAGCACCACAG
**hSDHA**	CAGCACAGGGAGGAATCAAT	TCACGGTGTCGTAGAAATGC
**hNDAUF10A**	TTCCTTCAACCTTGGTGGTT	CTGACCACAGGGCAGACC
**hCPT1A**	TGGAAAGTCTCAGCATATGGAA	CCAGCACATGAACTTGGTGA
**hCOX5A**	CAAAGTGTAAACCGCATGGAT	TCCAGGTAACTGTTCACACTCAA
**hFATP4**	AGCAGTGTAGCCAACTTCCT	CACGAACTCATTGCGGTTCT
**hDGAT2**	GTTTTTCTTGGGTGGCTGAT	CTGAGGCCACCAGTAAGCAT
**hATF6**	GAGTCATCGCGTCTCTCCAC	GGCCTCAGAGTTGACGGAAG
**hCHOP**	AAGGCACTGAGCGTATCATGT	TGAAGATACACTTCCTTCTTGAACA
**hACSL1**	AACAGACGGAAGCCCAAGC	TCGGTGAGTGACCATTGCTC

Mouse qRT PCR Primers

**Gene name**	Forward (5’-3’)	Reverse (5’-3’)
mAngptl1	TCTTCTCTGCTGCCCAC	GTGAGCCTCTGCACAATC

mAngptl2	GGGACCTTAACTGTGC	GAATGGCTACAGGTACCA
mAngptl3	GATGGCTCTGTCAATT	CATCAATGTTTCCAAACCC
mAngptl4	ACTTCAGATGGAGGCTGGAC	TCCGAAGCCATCCTTGTAGG
mAngptl5	GTCACCTAAGGCCAGATGCTT	TGCACTCCCTGACTTGCATT
mAngptl6	TATCCACCGGCTCACC	TCTGCGTAGCGTGCATTG
mAngptl7	CACCGTGAGGCATGTG	GGTTGGTCTTTATGAACTG
mAngptl8	GCTTTACACCTTCGAGCTGA	ATCCAGGTAGTCTCAGGCTG
mCPT1a	TTGATCAAGAAGTGCCGGACGAGT	GTCCATCATGGCCAGCACAAAGTT
mLdha	CCTGCACCAAACATGCCTA	CACTGGAGCCAGGTTATACGA
mSlc2a1	ATCATGGGCAATGCAGACTT	GTAGCAGGGCTGGGATGA
mHk2	GTTTGTACATTTATTTGCCCTCCT	AGCGTGCATCTCGTGTAAAA
mPfkfb3	CAGAGGACAGCTTGACAGGA	GAGGCCTGGGCTCAATCT
mAcsl1	TGGGGTGGAAATCATCAGCC	CACAGCATTACACACTGTACAACG
**m18S**	TTCTGGCCAACGGTCTAGACAAC	CCAGTGGTCTTGGTGTGCTGA
mAngtpl4 **Exon4**	GGGACTGCCAGGAACTCTTC	AATGGTGGAGACCCCAGAGG
mAngtpl4 **Exon5**	GAGGCTGGACAGTGATTCAGAGA	GGGATCTCCGAAGCCATCCTT

Genotyping Primers

	**Forward (5’-3’)**	**Reverse (5’-3’)**
Angptl4 Flox (genotyping)	TGTGGGGTAGGCTTTAGGTG	GCTTCTCTCCCTGAGCCTTT
ERT2 (genotyping)	ATACCGGAGATCATGCAAGC	ATGTGAACCAGCTCCCTGTC

Supplementary Table 2. List of siRNAs and shRNAs

siRNAs

ON-TARGETplus Human *ANGPTL4* siRNA (SMARTPool)	Horizon Discovery	Catalog # L-007807-00-0020
Accell Human *LIPG* siRNA (SMARTPool)	Horizon Discovery	Catalog # E-009601-00-00010
ON-TARGETplus Non-targeting Pool	Horizon Discovery	Catalog# D-001810-10-20
SMARTvector mouse *Angptl4* Lentiviral shRNA (set of 3)	Horizon Discovery	Catalog ID:V3SM11244-01EG57875<br>SET1: V3SM11241-233353210<br>Clone Id: V3SVMM01_13290866<br>SET2: V3SM11241-237020981<br>Clone Id: V3SVMM01_16958618<br>SET3: V3SM11241-231728343<br>Clone Id: V3SVMM01_11665979
SMARTvector Empty Vector Control	Horizon Discovery	Catalog ID:VSC11649

Supplementary Table 3: List of Antibodies

Antibodies

**Immunoblots**		
Phospho-AMPKα (Thr172) (40H9) Rabbit mAb	Cell Signaling Technology	Cat #2535
AMPKα (D5A2) Rabbit mAb	Cell Signaling Technology	Cat #5831
Phospho-p44/42 MAPK (Erk1/2) (Thr202/Tyr204) (D13.14.4E) XP® Rabbit mAb	Cell Signaling Technology	Cat #4370
p44/42 MAPK (Erk1/2) (137F5) Rabbit mAb	Cell Signaling Technology	Cat #4695
HSP90	BD Biosciences	Cat# 610419
VEGF Receptor 2 (55B11) Rabbit mAb	Cell Signaling Technology	Cat #2479
Hexokinase II (C64G5) Rabbit mAb	Cell Signaling Technology	Cat #2867
LDHA (C4B5) Rabbit mAb	Cell Signaling Technology	Cat #3582
PFKP (D4B2) Rabbit mAb	Cell Signaling Technology	Cat #8164
PKM2 (D78A4) XP® Rabbit mAb	Cell Signaling Technology	Cat #4053

PDH (C54G1) Rabbit mAb	Cell Signaling Technology	Cat #3205
ACSL1 (D2H5) Rabbit mAb	Cell Signaling Technology	Cat# 9189
CPT1A [8F6AE9] Mouse mAb	Abcam	Cat# ab128568
CD36 Rabbit pAB (Clone 948)	Proteintech	Cat# 18836-1-AP
NG2 (clone: 546930) mAb	Invitrogen	Cat# MA5-24247
**SECONDARY ANTIBODIES**		
Goat anti-Rabbit IgG, Alexa Fluor 800	Invitrogen	Cat# A32735
Goat anti-Mouse IgG, Alexa Fluor 800	Invitrogen	Cat# A32730
Goat anti-Mouse IgG, Alexa Fluor 680	Invitrogen	Cat# A21058
Goat anti-Rabbit IgG, Alexa Fluor 680	Invitrogen	Cat# A21109
**Immunofluorescence/Flowcytometry**		
CD31	BD Bioscience	Cat# 550274
CD31-PE, Clone MEC 13.3	BD Bioscience	Cat# 553373
CD31-BV421, Clone MEC 13.3	BD Bioscience	Cat# 562939
Ter119-FITC	BioLegend	Cat# 116205
α-SMA-eFluor™ 660	Invitrogen	Cat# 50-9760-82
Ki67-APC, Clone: 16A8	BioLegend	Cat# 652405
Ki67 PE	BioLegend	Cat# 652404
CD36-FITC	Invitrogen	Cat# 11-0369-42
CD36-APC	BioLegend	Cat# 102611
CD45-PE,	BioLegend	Cat# 157603

CD45-APC	BioLegend	Cat# 103112
Anti-BrdU-PE	eBioscience	Cat# 12-5071-41
Integrin alpha v beta 3	Invitrogen	Cat# 11-0519-41
Anti-ERG	Abcam	Cat# ab92513
