# Supplementary material for: Suppression of angiopoietin-like 4 reprograms endothelial cell metabolism and inhibits angiogenesis
Source: Nat Commun. 2023 Dec 12;14:8251. doi: 10.1038/s41467-023-43900-0 (PMC10716292; doi:10.1038/s41467-023-43900-0)
Supplement: Supplementary file 3 — Description of Additional Supplementary Files [file 41467_2023_43900_MOESM3_ESM.pdf]

DESCRIPTION OF ADDITIONAL SUPPLEMENTARY FILES

Suppression of Angiopoietin-like 4 Reprograms Endothelial Cell Metabolism and Inhibits Angiogenesis

Balkrishna Chaube[1,2,3], Kathryn M Citrin[1,2,3,4], Mahnaz Sahraei[1], Abhishek K. Singh[1,2], Diego Saenz de Urturi[1,2,3], Wen Ding[1], Richard W Pierce[2,5], Raaisa Raaisa[6], Rebecca Cardone[6], Richard Kibbey[3,4,6], Carlos Fernández-Hernando[1,2,3,7] and Yajaira Suárez[1,2,3,7*].

This file contains:

Supplementary Dataset Descriptions

File name: Supplementary Data 1

Description: Steady-state analysis of metabolites from glycolysis and the TCA cycle in HUVECs transfected with siRNA for ANGPTL4 or a non-silencing control siRNA (NS).

- Sheet 1: Raw metabolite data for HUVECs transfected with either NS or siRNA targeting ANGPTL4.

- Sheet 2: Normalized metabolite concentrations to total protein, located under the tab ‘Norm_Data’.

- Sheet 3: Total protein concentration data for each well from HUVECs transfected with either NS or siRNA targeting ANGPTL4.

- Sheet 4: The individual MRM transition pairs (Q1/Q3) are detailed in tab 4.

This dataset corresponds to Fig. 4h.
