# Supplementary material for: Suppression of angiopoietin-like 4 reprograms endothelial cell metabolism and inhibits angiogenesis
Source: Nat Commun. 2023 Dec 12;14:8251. doi: 10.1038/s41467-023-43900-0 (PMC10716292; doi:10.1038/s41467-023-43900-0)
Supplement: Supplementary file 5 — Reporting Summary [file 41467_2023_43900_MOESM5_ESM.pdf]

nature portfolio

Corresponding author(s): Dr. Yajaira Suarez

Last updated by author(s): 11-06-2023

Reporting Summary

Nature Portfolio wishes to improve the reproducibility of the work that we publish. This form provides structure for consistency and transparency in reporting. For further information on Nature Portfolio policies, see our Editorial Policies and the Editorial Policy Checklist.

Statistics

For all statistical analyses, confirm that the following items are present in the figure legend, table legend, main text, or Methods section.

n/a	Confirmed	
☐	☒	The exact sample size (*n*) for each experimental group/condition, given as a discrete number and unit of measurement
☐	☒	A statement on whether measurements were taken from distinct samples or whether the same sample was measured repeatedly
☐	☒	The statistical test(s) used AND whether they are one- or two-sided<br>*Only common tests should be described solely by name; describe more complex techniques in the Methods section.*
☐	☒	A description of all covariates tested
☐	☒	A description of any assumptions or corrections, such as tests of normality and adjustment for multiple comparisons
☐	☒	A full description of the statistical parameters including central tendency (e.g. means) or other basic estimates (e.g. regression coefficient) AND variation (e.g. standard deviation) or associated estimates of uncertainty (e.g. confidence intervals)
☒	☐	For null hypothesis testing, the test statistic (e.g. *F*, *t*, *r*) with confidence intervals, effect sizes, degrees of freedom and *P* value noted<br>*Give P values as exact values whenever suitable.*
☒	☐	For Bayesian analysis, information on the choice of priors and Markov chain Monte Carlo settings
☒	☐	For hierarchical and complex designs, identification of the appropriate level for tests and full reporting of outcomes
☒	☐	Estimates of effect sizes (e.g. Cohen's *d*, Pearson's *r*), indicating how they were calculated

Our web collection on statistics for biologists contains articles on many of the points above.

Software and code

Policy information about availability of computer code

Data collection	Western blot acquisition: Odyssey Infrared Imaging System (LI-COR Biotechnology)<br>Glucose and FA uptake, CD36 and Integrins surface expression experiments: BD FACS LSR-II; EVOS digital inverted florescence microscope (AMG), Leica SP5 II confocal microscope equipped with a 63X Plan Apo Lense.<br>RNA Seq analysis: Illumina HiSeq 2500. Agilent Bioanalyzer<br>Seahorse Xtracellular Flux analzyer (Agilent)<br>qRT-PCR: iCycler Real-Time Detection System (Biorad)<br>Mass spec: AB Sciex QTrap 5500.
Data analysis	Statistical analysis: GraphPad Prism Software Version 9.0<br>FACS analysis: FlowJo v10<br>Seahorse Wave Desktop Software (Agilent)<br>Fluorescent/Western blot image analysis/quantification: ImageJ (NIH)<br>RNAseq analysis: PartekFlow® software, version 8.0.19.0405 (Partek, Inc., St. Louis, MO), R studio, Ingenuity Pathway Analysis Spring Release 2019 (Ingenuity Systems QIAGEN.

For manuscripts utilizing custom algorithms or software that are central to the research but not yet described in published literature, software must be made available to editors and reviewers. We strongly encourage code deposition in a community repository (e.g. GitHub). See the Nature Portfolio guidelines for submitting code & software for further information.

Data

Policy information about availability of data

All manuscripts must include a data availability statement. This statement should provide the following information, where applicable:

- Accession codes, unique identifiers, or web links for publicly available datasets
- A description of any restrictions on data availability
- For clinical datasets or third party data, please ensure that the statement adheres to our policy

RNA-seq data that support the findings of this study have been deposited in the Gene Expression Omnibus under accession code GSE211128 (https://www.ncbi.nlm.nih.gov/geo/query/acc.cgi?acc=GSE211128).
All other data that support the findings of this study are within the article and its Supplementary Information, Supplementary Data1 and Source Data files.

Research involving human participants, their data, or biological material

Policy information about studies with human participants or human data. See also policy information about sex, gender (identity/presentation), and sexual orientation and race, ethnicity and racism.

Reporting on sex and gender	*Use the terms sex (biological attribute) and gender (shaped by social and cultural circumstances) carefully in order to avoid confusing both terms. Indicate if findings apply to only one sex or gender; describe whether sex and gender were considered in study design; whether sex and/or gender was determined based on self-reporting or assigned and methods used. Provide in the source data disaggregated sex and gender data, where this information has been collected, and if consent has been obtained for sharing of individual-level data; provide overall numbers in this Reporting Summary. Please state if this information has not been collected. Report sex- and gender-based analyses where performed, justify reasons for lack of sex- and gender-based analysis.*
Reporting on race, ethnicity, or other socially relevant groupings	*Please specify the socially constructed or socially relevant categorization variable(s) used in your manuscript and explain why they were used. Please note that such variables should not be used as proxies for other socially constructed/relevant variables (for example, race or ethnicity should not be used as a proxy for socioeconomic status). Provide clear definitions of the relevant terms used, how they were provided (by the participants/respondents, the researchers, or third parties), and the method(s) used to classify people into the different categories (e.g. self-report, census or administrative data, social media data, etc.) Please provide details about how you controlled for confounding variables in your analyses.*
Population characteristics	*Describe the covariate-relevant population characteristics of the human research participants (e.g. age, genotypic information, past and current diagnosis and treatment categories). If you filled out the behavioural & social sciences study design questions and have nothing to add here, write "See above."*
Recruitment	*Describe how participants were recruited. Outline any potential self-selection bias or other biases that may be present and how these are likely to impact results.*
Ethics oversight	*Identify the organization(s) that approved the study protocol.*

Note that full information on the approval of the study protocol must also be provided in the manuscript.

Field-specific reporting

Please select the one below that is the best fit for your research. If you are not sure, read the appropriate sections before making your selection.

☒ Life sciences ☐ Behavioural & social sciences ☐ Ecological, evolutionary & environmental sciences

For a reference copy of the document with all sections, see nature.com/documents/nr-reporting-summary-flat.pdf

Life sciences study design

All studies must disclose on these points even when the disclosure is negative.

Sample size	No sample size calculations were performed. Sample size was s determined to be adequate for each study was chosen and based on literature documentation of similar well-characterized experiments (Singh et al.,2021, Aryal et al., 2018, Chamorro-Jorganes et al., 2016; Sahraei et al 2019). Sample sizes for cell culture experiments (at least three biological replicates) were chosen based on experience working with HUVECs (Chamorro-Jorganes et al., 2016; Chamorro-Jorganes et al., 2014). Sample sizes are listed in the figure legends and main text.
Data exclusions	For siRNA experiments, knockdown was assessed prior to downstream analyses (gene expression, metabolic assays, metabolomics etc). Any individual experiments that did not achieve 75 % knockdown (by mRNA expression) was excluded from subsequent analyses. For all other in vitro experiments, no data was excluded. For tumor studies, mice with no palpable mass at days 6-8 were removed from the study.
Replication	In vitro experiments were routinely repeated independently and 3-6 biological replicates were used. Several cell culture experiments (knockdown of ANGPTL4 with different treatments, to assess EC cell functions, gene expression, cell proliferation, and metabolic assays) were replicated successfully by at least two investigators. Multiple mice (n = 3-10 per group) were used in all in vivo experiments. All

replication attempts were successful.

Randomization: Mice were weight-matched and randomized to experimental groups before the start of any experiments.

Blinding: When possible, blinding was performed during data collection and analysis for in vitro and in vivo experiments. For experiments in which investigators could not be blinded for practical reasons (i.e., for treatment of mice and cells), results were independently analyzed by different investigators.

Reporting for specific materials, systems and methods

We require information from authors about some types of materials, experimental systems and methods used in many studies. Here, indicate whether each material, system or method listed is relevant to your study. If you are not sure if a list item applies to your research, read the appropriate section before selecting a response.

Materials & experimental systems

n/a	Involved in the study
☐	☒ Antibodies
☐	☒ Eukaryotic cell lines
☒	☐ Palaeontology and archaeology
☐	☒ Animals and other organisms
☒	☐ Clinical data
☒	☐ Dual use research of concern
☒	☐ Plants

Methods

n/a	Involved in the study
☒	☐ ChIP-seq
☐	☒ Flow cytometry
☒	☐ MRI-based neuroimaging

Antibodies

Antibodies used: The following antibodies were used in this study: Phospho-AMPKα (Thr172) (Cell Signaling #2535, 1:1000), AMPKα (Cell Signaling #5831, 1:1000), HSP90 (BD Biosciences #610418, 1:1,000), Phospho-ERK1/2 (Cell Signaling #4370, 1:1000), ERK1/2 (Cell Signaling #4695, 1:1000), VEGF Receptor 2 (Cell Signaling # 2479, 1:1000), Hexokinase II (Cell Signaling #2867, 1:1000), LDHA (Cell Signaling #3582, 1:1000), PFKP (Cell Signaling #8164; 1:1000), PKM2 (Cell Signaling #4053, 1:1000), PDH (Cell Signaling #3205, 1:1000), ACSL1 (Cell Signaling #9189, 1:1000), CPT1A (Abcam #ab128568, 1:1000), CD36 (Proteintech #18836-1-AP, 1:1000), fluorescently labeled secondary antibodies (Invitrogen #A21058, #AA21109; 1:5000). For immunofluorescence studdies, the following antibodies were used- CD31 (BD Bioscience #550274, 1:100), CD31- PE, Clone MEC 13.3 (BD Bioscience #553373, 1:100), CD45-APC (BioLegend # 103112), Ter119-FITC (BioLegend #116205, 1:200), aSMA-eFluor™ 488 (Invitrogen # 53-9760-82, 1:100), Ki-67 eFluor™ 488 (Invitrogen #53-5698-82, 1:100), Ki67-APC, Clone: 16A8 (BioLegend # 652405), CD36-FITC (Invitrogen # 11-0369-42, 1:200), CD36-APC (BioLegend # 102611), Integrin alpha v beta 3 (Invitrogen # 11-0519-41, 1:200), ERG (Abcam #ab92513), (see Supplementary Table 3).

Validation: All antibodies from Cell Signaling, Abcam, BD Biosciences, Proteintech, BioLegend, and Invitrogen have been validated by their respective manufacturers for use in Western blotting, IHC, flow cytometry, and immunofluorescence. This validation information can be found on each manufacturer's website. We also performed aditional validation using positive and negetive controls. For antibodies used for Western analyses, positive control lysates from either cell lines or tissue sample known to express the protein were used for detection, and negative control lysate were used from cell line or tissue samples known not to express the protein for detection.
For flow cytometry/FACS, a range of appropriate negative and positive controls was used to verify specificity including cells only negative control (no primary Ab, no secondary Ab to check for background autofluoresence), primary Ab control (primary Ab, but no secondary to check for non-specific binding of primary), treated cells (secondary Ab, but no primary Ab to check for non-specific binding of secondary Ab on treated cells), isotype control (use isotype control primary Ab with secondary Ab to verify that the primary is specific and does not result of non-specific Fc receptor binding), compensation controls for each fluorochrome (positive control to set-up cyclometer alignment and to remove spectral overlap).

Eukaryotic cell lines

Policy information about cell lines and Sex and Gender in Research

Cell line source(s): Human umbilical vein endothelial cells (HUVECs), were obtained from Vascular Biology and Therapeutics (VBT) Core Facility at Yale university that routinely isolate these cells form discarded tissue (umbilical cords) with high standard internal controls. The mouse lung cancer cell line (LL/2 (LLC1-ATCC CRL-1642) and mouse melanoma cell line (B16-F0-ATCC CRL-6322) were purchased from ATCC

Authentication: All primary HUVECs were authenticated by the VBT core at Yale University and were used within 3-5 passages. This includes cell morphology and rate of cell division and expression of specific cell markers and response to TNFa or VEGFA stimulation. Cells were used within 6 total passages following receipt from the vendor or isolation. The stable cell lines, LLC and B16F10, were authenticated based on morphology checks at both high and low plating densities, and growth curve analyses.

Mycoplasma contamination	All cell lines tested negative for mycoplasma contamination.
Commonly misidentified lines (See ICLAC register)	No commonly misidentified cell lines were used in this study.

Animals and other research organisms

Policy information about studies involving animals; ARRIVE guidelines recommended for reporting animal research, and Sex and Gender in Research

Laboratory animals	Mice bearing a loxP-flanked Angptl4 allele (Angptl4loxP/loxP mice) were generated as described previously (Aryal et al., 2018). Endothelial-specific ANGPTL4–deficient mice (Cadh5-CreERT2; Angptl4loxP/loxP) were generated by breeding Cadh5-CreERT2; Angptl4loxP/+ mice with Angptl4loxP/+ mice. All mouse strains were in the C57BL6J genetic background. .All mice were housed in a barrier animal facility with a constant temperature and humidity in a 12-hour dark/light cycle. All mice were fed with a standard chow diet (CD) and water and food were provided ad libitum, temperature was between 20 and 24 °C and relative humidity between 45 and 65 rH. (see Method section for more details)
Wild animals	No wild animals were used in this study.
Reporting on sex	Both male and female mice were used since no sex-specific differences were observed. Female mice were mostly used for tumor angiogenesis experiments due to their reduced fighting behaviors which facilitates group housing, randomization and generation of matched cohorts.
Field-collected samples	No field-collected samples were used in this study.
Ethics oversight	Experiments were conducted under the ethical guidelines and protocols approved by IACUC (Institutional Animal Care and Usage Committee) in Yale University School of Medicine (Animal protocol no 2022-116576)

Note that full information on the approval of the study protocol must also be provided in the manuscript.

Plants

Seed stocks	*Report on the source of all seed stocks or other plant material used. If applicable, state the seed stock centre and catalogue number. If plant specimens were collected from the field, describe the collection location, date and sampling procedures.*
Novel plant genotypes	*Describe the methods by which all novel plant genotypes were produced. This includes those generated by transgenic approaches, gene editing, chemical/radiation-based mutagenesis and hybridization. For transgenic lines, describe the transformation method, the number of independent lines analyzed and the generation upon which experiments were performed. For gene-edited lines, describe the editor used, the endogenous sequence targeted for editing, the targeting guide RNA sequence (if applicable) and how the editor was applied.*
Authentication	*Describe any authentication procedures for each seed stock used or novel genotype generated. Describe any experiments used to assess the effect of a mutation and, where applicable, how potential secondary effects (e.g. second site T-DNA insertions, mosiacism, off-target gene editing) were examined.*

Flow Cytometry

Plots

Confirm that:

- [x] The axis labels state the marker and fluorochrome used (e.g. CD4-FITC).
- [x] The axis scales are clearly visible. Include numbers along axes only for bottom left plot of group (a 'group' is an analysis of identical markers).
- [x] All plots are contour plots with outliers or pseudocolor plots.
- [x] A numerical value for number of cells or percentage (with statistics) is provided.

Methodology

Sample preparation	HUVECs were analyzed for surface expression, glucose/FA uptake, cell viability- AnnexinV/PI, and proliferation- EdU incorporation by fluorescence flow cytometry. After desired culture conditions, cells monolayers were washed twice in PBS, then trypsinized and washed with cold 1% BSA/PBS, centrifuged, and washed again in cold 1% BSA/PBS. After desired staining conditions, cells were washed twice with cold PBS and analyzed on a FACS LSR-II flow cytometer (BD Biosciences) using Flow Jo analysis software collecting 10,000 cells gated viable cells per sample. In all cases negative controls for staining were used. For Flow cell sorting of MLECs, live cells were gated by staining with LIVE/DEAD™ Fixable Blue Dead Cell Stain Kit (Invitrogen, Cat# L23105,). All antibodies were titrated to determine optimal signal-to-noise separation and minimize background fluorescence. The gating strategy was set up using Fluorescence Minus One (FMO) controls.
Instrument	BD FACS LSR-II and BD FACS Aria (BD Biosciences)

Software	BD FACS DIVA and Flow Jo v10
Cell population abundance	Live cells were enriched using FSC/SSC. 10,000 events were collected for each sample.
Gating strategy	Live cells were gated using FSC and SSC. Compensation controls was used for each fluorochrome (positive control to setup cytometer alignment and to remove spectral overlap). Cells incubated with negative or isotype controls were used to set positivity for (Probes). The results are expressed in terms of specific geometric mean intensity of fluorescence (M.I.F.) after subtracting fluorescence of cells incubated with isotype or negative control respectively. The gating strategy was set up using Fluorescence Minus One (FMO) controls.

☒ Tick this box to confirm that a figure exemplifying the gating strategy is provided in the Supplementary Information.
